# Activation of the EIF2α/ATF4 and ATF6 Pathways in DU-145 Cells by Boric Acid at the Concentration Reported in Men at the US Mean Boron Intake

**DOI:** 10.1007/s12011-016-0824-y

**Published:** 2016-09-01

**Authors:** Sarah E. Kobylewski, Kimberly A. Henderson, Kristin E. Yamada, Curtis D. Eckhert

**Affiliations:** 1 Interdepartmental Program in Molecular Toxicology, University of California, Los Angeles, CA USA; 2 Department of Environmental Health Sciences, University of California, Fielding School of Public Health, 650 Charles E. Young Dr., Los Angeles, CA 90095 USA

**Keywords:** ATF4, ATF6A, Boric acid, Boron, Calreticulin (CALR), EDEM1, eIF2α (EIF2A), GADD34 (PPP1R15A), GADD153/CHOP (DDIT3), GRP78/BiP (HSPa5), GRP94 (HSP90B1), Herp (HERPUD1), Hrd1 (SYVN1), RE1 (ERN1), XBP1, DU-145, Nutrition

## Abstract

Fruits, nuts, legumes, and vegetables are rich sources of boron (B), an essential plant nutrient with chemopreventive properties. Blood boric acid (BA) levels reflect recent B intake, and men at the US mean intake have a reported non-fasting level of 10 μM. Treatment of DU-145 prostate cancer cells with physiological concentrations of BA inhibits cell proliferation without causing apoptosis and activates eukaryotic initiation factor 2 (eIF2α). EIF2α induces cell differentiation and protects cells by redirecting gene expression to manage endoplasmic reticulum stress. Our objective was to determine the temporal expression of endoplasmic reticulum (ER) stress-activated genes in DU-145 prostate cells treated with 10 μM BA. Immunoblots showed post-treatment increases in eIF2α protein at 30 min and ATF4 and ATF6 proteins at 1 h and 30 min, respectively. The increase in ATF4 was accompanied by an increase in the expression of its downstream genes growth arrest and DNA damage-induced protein 34 (GADD34) and homocysteine-induced ER protein (Herp), but a decrease in GADD153/CCAAT/enhancer-binding protein homologous protein (CHOP), a pro-apoptotic gene. The increase in ATF6 was accompanied by an increase in expression of its downstream genes GRP78/BiP, calreticulin, Grp94, and EDEM. BA did not activate IRE1 or induce cleavage of XBP1 mRNA, a target of IRE1. Low boron status has been associated with increased cancer risk, low bone mineralization, and retinal degeneration. ATF4 and BiP/GRP78 function in osteogenesis and bone remodeling, calreticulin is required for tumor suppressor p53 function and mineralization of teeth, and BiP/GRP78 and EDEM prevent the aggregation of misfolded opsins which leads to retinal degeneration. The identification of BA-activated genes that regulate its phenotypic effects provides a molecular underpinning for boron nutrition and biology.

**Electronic supplementary material:**

The online version of this article (doi:10.1007/s12011-016-0824-y) contains supplementary material, which is available to authorized users.

## Background

Boron (B) intake is associated with reduced risk of prostate cancer in men [1–5], lung cancer in women [6], improvement in executive brain function [7], bone mineralization and strength [8–10], and a decrease in inflammation in animals [11]. Plants require boron as an essential nutrient and are the major source of boron in the human diet [12]. Greater than 90 % of dietary boron is absorbed and distributed to tissues as boric acid (BA) (WHO 1998) with 98 % eliminated in the urine after 120 h (Jansen et al. 1984). A study of men working in a large boric acid borax production plant found no differences in boron blood levels between workers with high and low occupational [13] exposure at the beginning of their 5-day work week [13]. Monday pre-shift non-fasting blood BA levels averaged 9.7 μM in these workers whose average dietary intake of 1.35 mg B/day was near the mean US intake of 1.42 mg/day for men over 30 years of age [12].

Human prostate cells were developed as a model for investigating the molecular cell biology of BA after epidemiological evidence showed that boron was associated with reduced risk of prostate cancer [3, 5]. Treatment of LNCaP and DU-145 prostate cancer cells with physiological concentrations or BA inhibits cell proliferation in a dose-dependent manner [5] without causing apoptosis but shifts them into a senescent-like phenotype [5, 14]. BA binds to cADPR and inhibits cADPR-activated Ca^2+^ release from the endoplasmic reticulum (ER) in a dose-dependent manner [15, 16] and lowers ER luminal Ca^2+^ concentrations. Low ER luminal Ca^2+^ induces phosphorylation of eIF2α, a major regulator of cellular responses to environmental stress [17]. In MEF cells, low activation of eIF2α activates the transcription factor ATF4 which induces genes in the integrated stress response, but not the CCAAT/enhancer-binding protein homologous protein (CHOP), an apoptotic gene, whereas high activation induces CHOP. Phosphorylation of eIF2α occurs following BA treatment of DU-145 and LNCaP prostate cells [18–20] and yeast [21]. In humans, blood levels of BA are dynamic, rising rapidly after a meal with an elimination half-life from 4 to 27.8 h depending on dose [22, 23]. Here, we treated DU-145 cells with a constant BA concentration of 10 μM, the level reported in men at the mean US boron dietary intake, and measured the temporal response of genes in the ER stress pathways.

## Methods

### Chemical

DMSO, DTT, boric acid, NaCl, Tris, methanol, MgCl_2_, sucrose, and methanol were purchased from Sigma-Aldrich (St. Louis, MO). Triton X-100, Tween-20, NP40, and cycloheximide were purchased from Fisher Scientific (Pittsburg, PA). Paraformaldehyde was purchased from Affymetrix/USB Corporation (Cleveland, OH). Thapsigargin and BSA were purchased from Santa Cruz Biotechnology (Santa Cruz, CA). Phosphatase inhibitors and protease were purchased from Calbiochem (San Diego, CA), and fetal bovine serum (FBS) was purchased from Gibco-Life Sciences (Grand Island, NY).

### Cell Culture

DU-145 prostate cancer cells were purchased from the American Type Culture Collection (Manassas, VA) and sub-cultured at a ratio of 1:6 and used for seven passages. The ATCC description of the DU-145 karyotype is given in Supplement 2 [24]. DU-145 is included in the COSMIC Cell Line Project, and available information for each gene measured in this paper is given in Tables 1-6 of Supplement 2. The cell culture medium was RPMI-1640 Medium (Gibco-Life Technologies, Grand Island, NY) supplemented with 10 % FBS, l-glutamine (200 mM), streptomycin (100 μg/mL), and penicillin (100 U/mL) (Gemini Bio Products, Sacramento, CA). Cells were plated on 10- or 15-cm plates (Corning Life Sciences, Corning, NY) and incubated in a humidified chamber at 37 °C and 5 % CO_2_ and 95 % air to 80 % confluency. Media used for all treatment groups were first stripped of boron by shaking with 2 g of Amberlite IRA 743 exchange resin (Sigma-Aldrich) for 12 h at 4 °C. H_2_O or BA (10 mM) was added respectfully to prepare untreated and 10 μM BA cell culture media [25]. Cells were cultured in untreated media until 80 % confluency and sub-cultured for experiments using untreated media or media adjusted to 10 μM BA.

### Polysome Profile

Polysome profiles were developed from untreated and BA-treated cells obtained from paired culture plates and processed in parallel. Plated cells were incubated in culture media containing 50 μg/mL cycloheximide for 10 min at 37 °C. Plates were then chilled on ice, the media removed, and cells were rinsed two to three times with ice-cold PBS containing 50 μg/mL cycloheximide. The cells were lysed in 500 μl lysis buffer (20 mM Tris (pH 7.5), 100 mM NaCl, 10 mM MgCl_2_, 0.4 % NP-40, 50 μg/mL cycloheximide, and protease and phosphatase inhibitors. The lysate was scraped with a spatula (Corning) and transferred to a microcentrifuge tube. The lysate was passed through a sterile 23-gauge needle (BD, Franklin Lakes, NJ) 8–10 times and incubated on ice for 10 min. The lysate was centrifuged at 8000×*g* for 10 min, and the supernatant was used for the polysome profile. The gradient was prepared by filling a SW41 centrifuge tube approximately halfway with 10 % gradient solution [20 mM Tris–HCl, pH 7.5, 100 mM NaCl, 5 mM MgCl_2_, 0.5 mM DTT, 0.1 mg/mL cycloheximide, and 10 % sucrose (*w*/*v*)]. A 23-gauge needle was used to add solution containing 20 mM Tris–HCl, pH 7.4, 100 mM NaCl, 5 mM MgCl_2_, 0.5 mM DTT, 0.1 mg/mL cycloheximide, and 50 % (*v*/*w*) sucrose to the bottom of the tube to form the lower layer. A gradient maker was used to create a 10–50 % gradient. Lysate with OD of 10 (up to 800 μl) was gently added to the top of the gradient. The tubes were balanced, gently placed in a pre-cooled SW41 rotor (Beckman Coulter, Brea, CA), and centrifuged for 3 h at 35,000 rpm. Fifty fractions were collected through a 23-gauge needle inserted through the bottom of the tube and immediately placed on ice. The absorbance of each fraction was measured at 254 nm. Monosomes and polysomes were quantified by measuring the area under the 80S monosomal peak and the area under the polysomes using the trapezoidal area under the curve method.

### Immunoblot Analysis

DU-145 cells were grown to 80 % confluency on 15-cm plates (Corning) and treated with 10 μM BA, 1 μM thapsigargin, or DMSO positive control vehicle for various times. Cells were washed with ice-cold phosphate buffer solution (PBS) supplemented with 0.1 % Tween (PBST) and treated with 100 μl radioimmunoprecipitation assay (RIPA) lysis buffer. Actin and GAPDH were used as loading controls and selected based on their molecular mass band separation from the protein of interest. Cells were scraped from plates using a spatula (Corning) placed on ice, and the lysate was passed through a 23-gauge needle (BD) 8–10 times. The protein was quantitated using Coomassie Plus Protein Assay (Thermo-Scientific, Waltham, MA). Aliquots containing 30–35 μg protein sample were separated using a 4–12 % gradient TGX SDS-PAGE (Bio-Rad, Hercules, CA) at 200 V for 30 min. Protein was transferred to a nitrocellulose membrane using a transfer buffer containing 20 % methanol at 40 V for 1.5 h. Membranes were blocked in 3 % BSA with 37.5 mM Tris (pH 8.8), 0.1 % Tween 20, and 125 mM NaCl for at least 4 h. Antibodies were selected from commercial suppliers based on their specificity for human orthologs and validated by comparison of immunoblot bands to standard molecular mass ladders (Bio-Rad). Antibody dilutions were selected based on testing of lots prior to use in experiments and ranged from 1:200 to 1:1000. Blocked membranes were incubated with the primary antibody for 1 h in PBST or 3 % BSA blocking solution and washed in PBST. They were then incubated with a secondary antibody with an HRP tag, followed by washing three times with PBST. The membranes were exposed to ECL Plus (Amersham/GE Healthcare, Pittsburg, PA) for 2–5 min and imaged using a Typhoon 9410 Variable Mode Imager (Amersham). Densitometry was performed using ImageQuant 5.2 software (Molecular Dynamics, Pittsburg, PA). All secondary antibodies were purchased from Santa Cruz Biotechnologies (Santa Cruz, CA). The following primary antibodies from Santa Cruz Biotechnology were used: GRP78/BiP (mouse monoclonal), Actin (goat polyclonal), GAPDH (mouse monoclonal), ATF4 (rabbit polyclonal), GADD34 (rabbit polyclonal), CHOP/Gadd153 (rabbit polyclonal), XBP-1 (rabbit polyclonal), and ATF6α (rabbit polyclonal). The eIF2α (rabbit polyclonal) and ph-eIF2α (rabbit polyclonal) antibodies were purchased from Cell Signaling (Danvers, MA), and ATF6 (mouse monoclonal) was purchased from Imgenex (San Diego, CA).

### TaqMan Real-Time PCR

DU-145 cells were grown on 10-cm plates (Corning) to 80 % confluency at least 24 h prior to treatment. Cells were treated with 10 μM BA, 1 μM thapsigargin, or DMSO vehicle for varying time points. RNA was isolated from cells at indicated time points (0, 0.25, 0.5, 1, 2, 3, 4, 5, 6, 12, or 24 h) using an RNeasy Mini Kit (Qiagen, Valencia, CA). Total RNA (2 μg) was reverse transcribed using Superscript III Reverse Transcriptase (Invitrogen) with random hexamer primers (Invitrogen) at a final volume of 20 μl at 25 °C, 10 min (10:00); 50 °C, 45:00; and 70 °C, 15:00. Applied Biosystems (ABI, Foster City, CA) TaqMan pre-designed assays were used for all genes as well as GAPDH as a control internal housekeeping gene. Plates were read by a 7500 Fast Real-Time PCR System using the 7500 Fast System Software v1.4.0 (ABI). Quantitation of gene expression levels was calculated from a standard curve created from reactions containing a combination of complementary DNA (cDNA) from all treatments for each gene.

### Immunofluorescent Microscopy

DU-145 cells were grown to 80 % confluency on glass coverslips (Fisher Scientific, Pittsburg, PA) and treated with either BA-free media, 10 μM BA, or 1 μM thapsigargin. Antibodies were tested on immunoblots of lysed cells prior to using them for immunohistochemistry. Cells stained for ATF6 were first fixed with 4 % paraformaldehyde in PBS and permeabilized with 0.5 % Triton X-100 in PBS. Fixed cells were blocked with 10 % FBS in PBS overnight. The next day, they were moved to a humidity chamber and incubated with anti-ATF6 (Imgenex, San Diego, CA) monoclonal antibody at a concentration of 1:50, followed by secondary Alexa 488 or FITC at 1:100. Coverslips were mounted with a mixture of Vectashield with 4′,6-diamidino-2-phenylindole (Vector Laboratories, Burlingame, CA) and regular Vectashield HardSet (Vector Laboratories) mounting mediums at 1:5. Images were captured with an Olympus DP72 camera (Olympus America, Center Valley, PA) connected to an Olympus BX51 fluorescence microscope (Olympus America) using an Olympus UIS2 UPlanFLN 100X/1.30 OilPh3 objective (Olympus America) and FITC and DAPI filters. Olympus DP2-BSW (Olympus America) or Adobe Photoshop (Adobe Systems Incorporated, San Jose, CA) software was used to merge and crop images.

### XBP1 Cleavage Analysis

Total RNA was extracted from BA (0–250 μM)-treated or thapsigargin (10 μM)-treated DU-145 cells using RNeasy Mini Kit (Qiagen). RNA was reverse transcribed using SuperScript III Reverse Transcriptase (Invitrogen). XBP1 cDNA was amplified with GoTaq Flexi DNA Polymerase (Promega, Madison WI) using the forward primer 5ʹ-CACCTGAGCCCCGAGGAG-3ʹ and reverse primer 5ʹ-TTAGTTCATTAATGGCTTCCAGC-3ʹ [HGNC:12801; NCBI Reference Sequence: NM_005080.3]. Fifty-microliter PCR reactions were run under the following amplification conditions: 95 °C for 2 min; 95 °C for 30 s, 60 °C for 30 s, 72 °C for 30 s, all for 25 cycles; and 72 °C for 5 min [26]. PCR products were run on 2 % agarose E-gels with SYBR Safe (Invitrogen) or 4 % ethidium bromide agarose gels (Fig. 11).

### Statistical Analysis

SigmaStat 3.1 (Systat Software, Point Richmond, CA) was used for analysis of data and selection of appropriate statistical tests. Results of the statistical analysis for each figure are provided in Supplement 1 Polysome profiles were developed from untreated and BA-treated cells obtained from paired culture plates processed in parallel. Monosomes and polysomes were quantified by measuring the area under the 80S monosomal peak and the area under the polysomes using the trapezoidal area under the curve method. The difference in means of polysome/monosome ratios from three independent experiments run on different days was evaluated using a paired *t* test. Replicates of data in Figs. 2, 3, 4, 5, 6, 7, 8, 9, 10, 11, and 12 represent cells plated from different seedings on different days. Analysis of timed studies used a one-way repeated measures analysis of variance to determine the effect of treatment time. Differences between the mean at pre-treatment time zero (control) and means at post-treatment time points were evaluated using the Holm–Sidak multiple comparison test. The number of replicates for each significant time point is given in the figure legends and Supplement 1. ImageJ software (NIH, Bethesda, MD) was used for immunofluorescence quantitation of stained cells. Only cells with clear borders of the ER and nucleus were selected for study. Mean intensity of nuclear and cytoplasmic areas of identical size was determined using the histogram tool in RGB mode. For ATF6 images, the polygon tool was used to trace the outer edges of the FITC and DAPI-stained areas and the ER was calculated by subtracting the area of the DAPI from the area of the FITC. Proof that DU-145 cells were capable of activating the genes under study was obtained by treatment with 1 μM thapsigargin, a strong inducer of ER stress that activates apoptosis [27]. The statistical significance of thapsigargin treatment in Figs. 2, 3, 4, 6, 8, 9, 10, and 12 was compared to treatment with its solvent DMSO using a *t* test.

### Availability of Supporting Data

Supplement 1 contains tables of statistical evaluations of the data for each figure. Supplement 2 gives the ATCC karyotype description of DU-145 and mutation status of genes available in COSMIC and Ensemble in Tables 1–6 [28].

## Results

### BA Causes a Decrease in the Polysome/Monosome Ratio

**Fig. 1:**
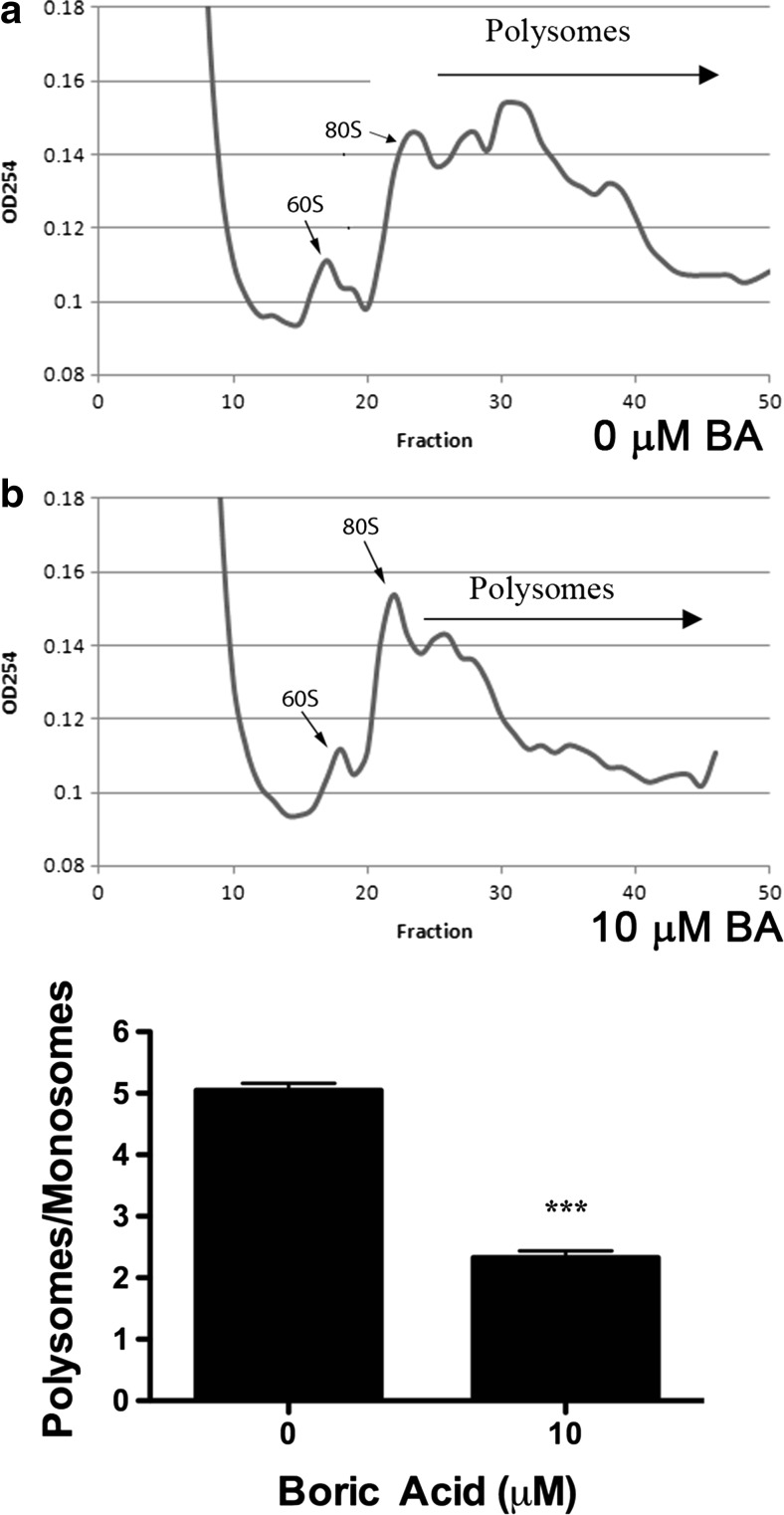
BA induces a lower polysome/monosome ratio in DU-145 cells indicating a reduction in global protein translation. DU-145 cells treated with 10 μM BA for 2 h (**a**) had a significantly lower polysome/monosome ratio than did DU-145 cells treated with 0 μM BA for 2 h (**b**). Polysomes from untreated and BA-treated cells were obtained from cells treated in parallel using paired culture plates and centrifuging together in the same rotor. Figure shows a representative replicate. The difference in polysome/monosome ratios from three independent experiments was evaluated using a paired *t* test (*p* < 0.001, *n* = 3)

Environmental conditions that stress ER function elicit a response that inhibits global translation and selectively enhances the transcription and translation of proteins needed to alleviate the stress [29]. Transcripts that are not translated form ribonuclear protein particles (mRNP) that sediment at 20S to 35S. Messenger RNAs (mRNAs) that are translated accumulate as 80S monosomes composed of small (40S) and large (60S) ribosomal subunits that travel along the mRNA during translational elongation. During protein synthesis, many monosomes can initiate translation on a single mRNA transcript to form a polyribosome or polysome. Polysomes disassemble under environmental conditions that inhibit protein synthesis, and this results in a decrease in the polysome/monosome ratio [30, 31]. We measured the areas under the 80S monosomal peak and polysomes using the trapezoidal area under the curve method. We show that treatment of DU-145 cells with 10 μM BA reduced the polysome/monosome ratio by approximately 54 %, demonstrating that protein translation was significantly inhibited, but not stopped (Fig. 1, Supplement 1).

### BA Induces Phosphorylation of eIF2α

**Fig. 2:**
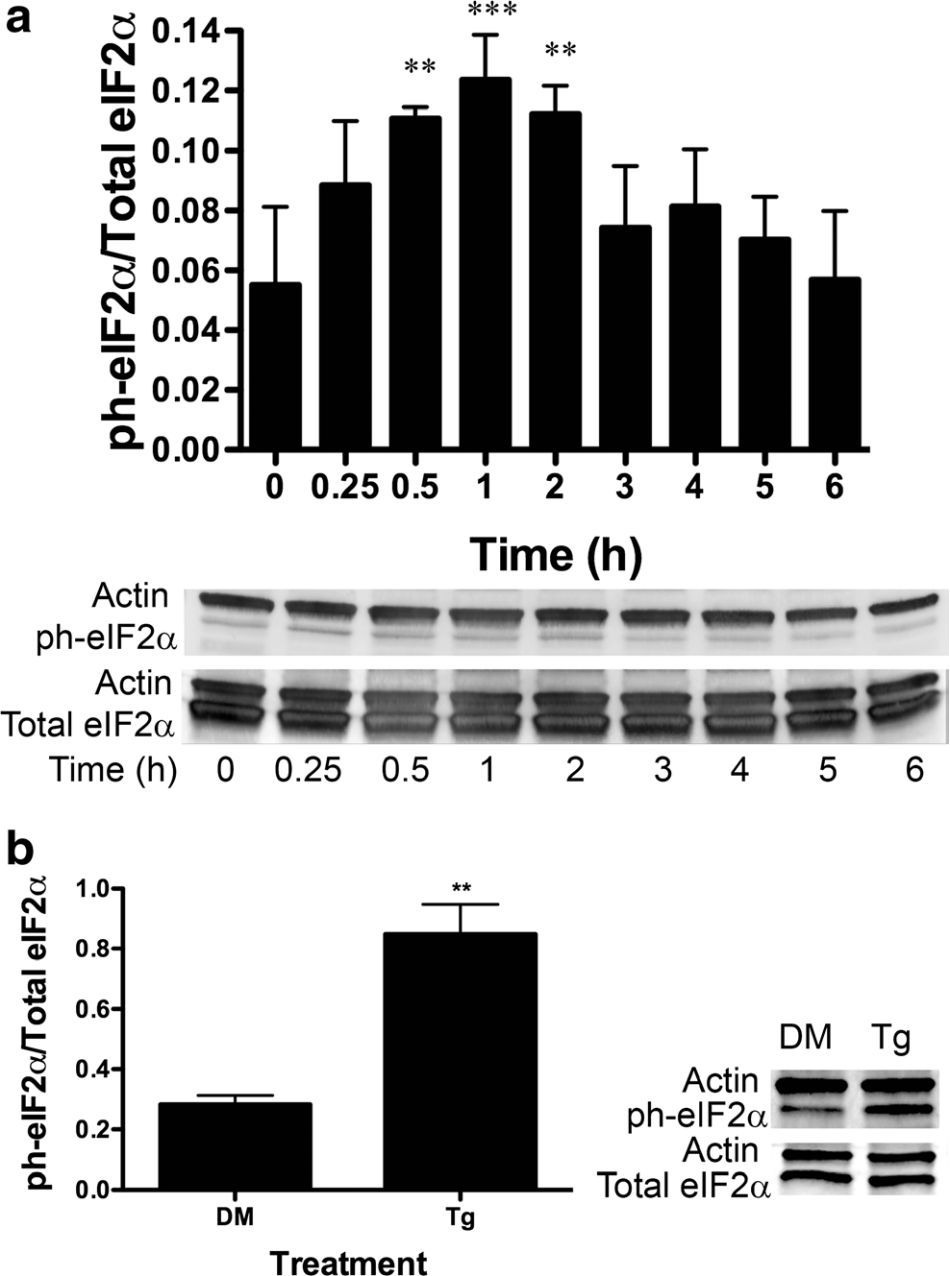
BA induces eIF2α phosphorylation at ser-51 in DU-145 cells. **a** DU-145 cells were treated with 10 μM BA for 0, 0.25, 0.5, 1, 2, 3, 4, 5, and 6 h. Phosphorylation of eIF2α was significantly higher at 0.5 (*p* < 0.006, *n* = 3), 1, (*p* < 0.001, *n* = 3), and 2 h (*p* < 0.005, *n* = 3) post-treatment. **b** DU-145 cells were treated with 1 μM thapsigargin (Tg), a strong positive control that induces stress and apoptosis, or DMSO (DM), the vehicle for Tg for 1 h, *p* < 0.01, *n* = 3. In Figs. 2, 3, 4, 5, 6, 7, 8, 9, 10, 11, and 12, the probabilities of statistical differences are represented as *p < 0.05, **p < 0.01, and ***p < 0.001. Gels shown in Figs. 2, 3, 4, 5, 6, 7, 8, 9, 10, 11, and 12 are a representative replicates of timed studies. Timed study data were analyzed using a one-way repeated measures analysis of variance (ANOVA) followed by a multiple comparison of individual post-treatment time points to treatment time 0 (control). Supplement 1 contains the ANOVA table for each figure giving the number of replicates for each time point and the results of the multiple comparison of each post-treatment time point to treatment time 0

The eIF2 complex is part of a large ternary complex (eIF2–GTP–tRNAi Met) that positions the initiator methionine at the first codon of mRNA to commence translation and protein synthesis. eIF2α is the regulatory subunit of the eIF2 complex, and phosphorylation on serine 51 inhibits the formation of the eIF2–GTP–tRNAi Met complex preventing the initiation of translation [32]. We measured phosphorylation of eIF2α at serine 51 in DU-145 cells treated with 10 μM BA. The proportion of eIF2α phosphorylated (eIF2α phosphorylation/total eIF2α) was mildly but significantly increased following 0.5, 1, and 2 h of treatment (Fig. 2a). The maximum increase in phosphorylation was 125 % at 60 min after BA treatment. As a positive control, we treated DU-145 cells with 1 μM thapsigargin or DMSO (vehicle) for 1 h. Thapsigargin, a strong inducer of ER stress [27], significantly induced phosphorylation of eIF2α in DU-145 cells (Fig. 2b).

### BA Induces GADD34 Transcription and Translation

**Fig. 3:**
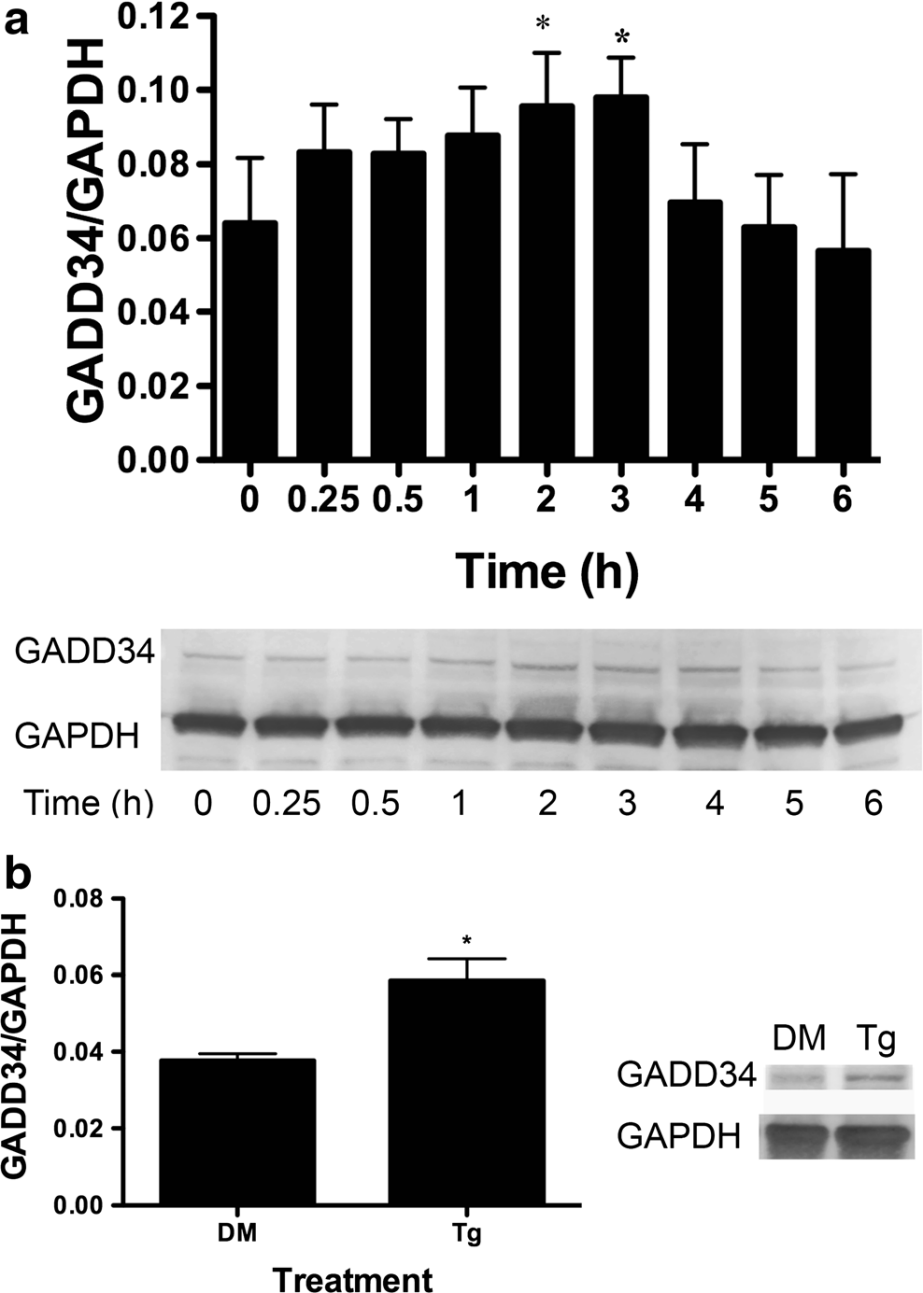
BA induces an increase in GADD34 protein in DU-145 cells. **a** DU-145 cells were treated with 10 μM BA for 0, 0.25, 0.5, 1, 2, 3, 4, 5, and 6 h. GADD34 was increased in cells at 2 (*p* = 0.018, *n* = 3) and 3 h (*p* = 0.011, *n* = 3). **b** DU-145 cells treated with 1 μM thapsigargin (Tg) or DMSO (DM) for 1 h, (*p* < 0.05, *n* = 3). The gel shown is a representative replicate. A description of the statistical analysis is given in the legend of Fig. 2

GADD34 associates with protein phosphatase 1 and promotes dephosphorylation of eIF2α [33]. BA treatment resulted in a maximum increase in mRNA of 125 % at 2 h and increase of protein of 53 % at 2 h (Figs. 7a and 3a, Table 1). Treatment with thapsigargin also increased GADD34 protein (Fig. 3b).

**Table 1:** Time of maximum response of untreated cells (0 min) to treatment with 10 μm boric acid

Gene (symbol(s))	Measured response; maximum change; post-treatment time^a^
Eukaryotic translation initiation factor (EIF2A)	Phosphorylation; +125 %; 60 min
Activating transcription factor 4 (ATF4)^b^	mRNA; +46 %; 2 h Protein; +53 %; 3 h
ATF4-activated genes	
Growth arrest and DNA damage-inducible protein 34 (GADD34); Protein phosphatase 1 regulatory subunit 15A (PPP1R15A)	mRNA; +125 %; 2 h Protein; +53 %; 2 h
DNA damage inducible transcript 3 (CHOP; GADD153; DDIT3)	mRNA; −59 %; 12 h Protein; −51 %; 3 h
Homocysteine-inducible, endoplasmic reticulum stress-inducible, ubiquitin-like domain member 1 (HERP, HERPUD1)	mRNA; +56 %; 4 h
Activating transcription factor 6A (ATF6A)^c^	Cleavage p70/p100; +77 %; 30 min Nuclear/cytoplasm staining; +29 %; 2 h
ATF6A-activated genes	
Heat shock protein family A (Hsp70) member 5 (GRP78; BiP; HSPA5)	mRNA; +149 %; 30 min Protein; +98 %; 60 min
ER degradation enhancer, mannosidase alpha-like 1 (EDEM1)	mRNA; +110 %; 24 h
Heat shock protein 90-kDa beta family member 1 (GRP94; HSP90B1)	mRNA no change
Calreticulin (CALR)	mRNA; +52 %; 8 h
X-box binding protein 1 (XBP1)	mRNA; +147 %; 24 h
Endoplasmic reticulum to nucleus signaling 1 (IRE1; ERN1)^c^	No change or negative change
IRE1-activated genes	
X-box binding protein 1 spliced (XBP1^s^)	Spliced XBP1 (XBP1s) was not observed at 0–250 μM BA
Synoviolin 1 (HRD1; SYVN1)	mRNA; −34 %; 24 h

^a^Maximum change from treatment with 10 μM boric acid was calculated using the mean of untreated cells (0 min) and the highest mean value post-treatment (data available in Supplement 1). Percent (%) change = [highest post-treatment value − pre-treatment value (at 0 min)] ÷ [pretreatment value (at 0 min)]. Note that the first significant change may have occurred earlier; see figures

^b^ATF4 is activated by low levels of EIF2α subsequent to EIF2α phosphorylation

^c^Stress sensor in the unfolded protein response stress pathway (UPR)

### BA Increases GRP78/BiP Transcription and Translation

**Fig. 4:**
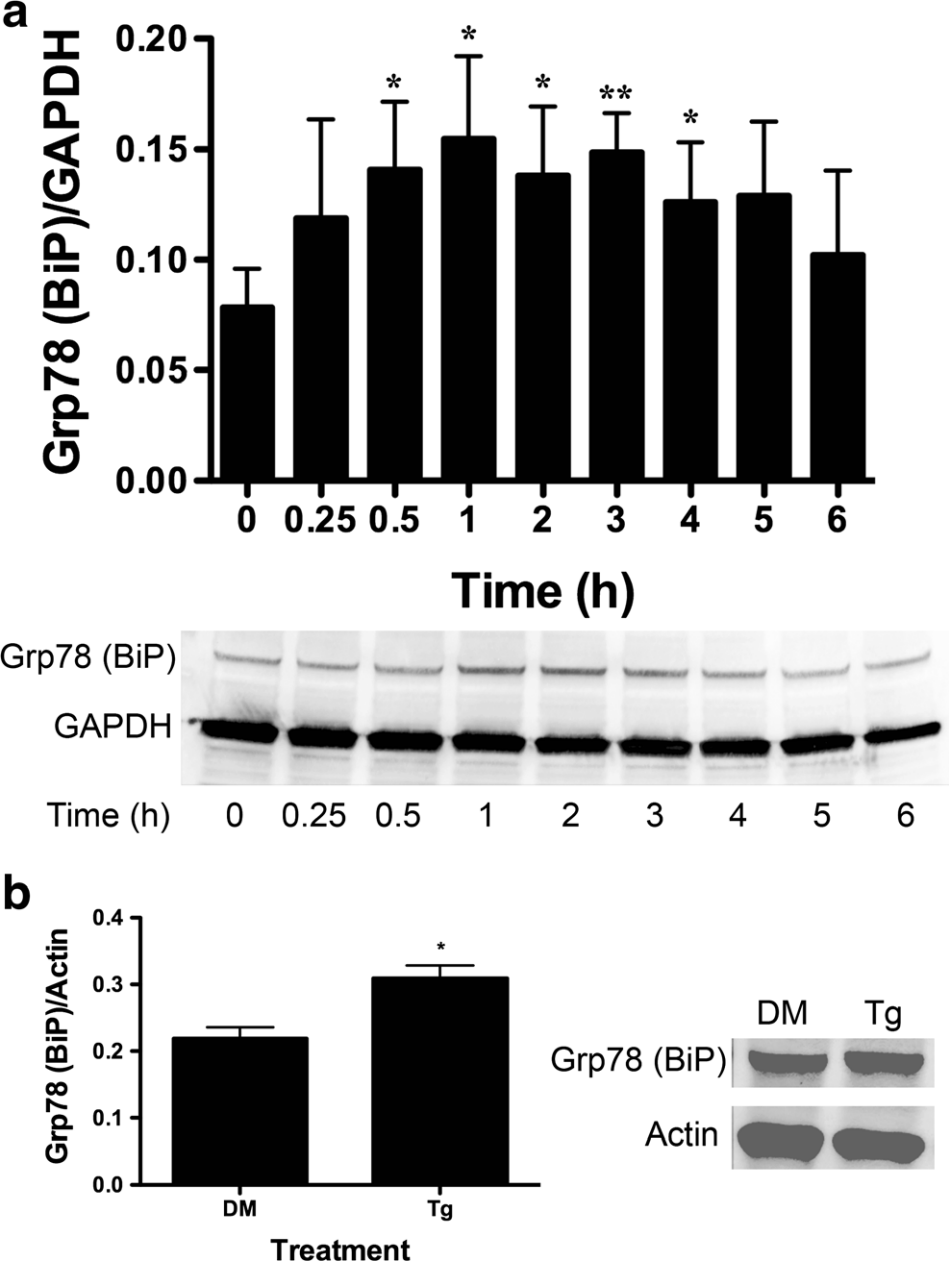
BA induces an increase in GRP78 (BiP) protein in DU-145 cells. **a** DU-145 cells were treated with 10 μM BA for 0, 0.25, 0.5, 1, 2, 3, 4, 5, and 6 h. GAPDH was used as an internal loading control. GRP78/BiP translation was increased 0.5 (*p* = 0.028, *n* = 4), 1 (*p* = 0.007, *n* = 4), 2 h (*p* = 0.032, *n* = 4), and 3 h (*p* = 0.013, *n* = 4). **b** DU-145 cells treated with 1 μM thapsigargin (Tg) or DMSO (DM) vehicle for 1 h (*p* < 0.05, *n* = 3). The gel shown is a representative replicate. A description of the statistical analysis is given in the legend of Fig. 2

Glucose-regulated protein (GRP78/BiP) is a major Ca^2+^-binding protein in the ER and binds to and maintains the three unfolded protein response (UPR) transmembrane sensors ATF6, PERK, and IRE1 in an inactive form [34, 35]. We measured GRP78/BiP in DU-145 cells treated with 10 μM BA. The maximum increase of mRNA was 149 % at 30 min and of protein 98 % at 60 min following BA treatment (Figs. 10a and 4a, Table 1). DU-145 cells treated with thapsigargin also increased GRP78/BiP protein (Fig. 4b).

### BA Induces ATF4 Transcription and Translation

**Fig. 5:**
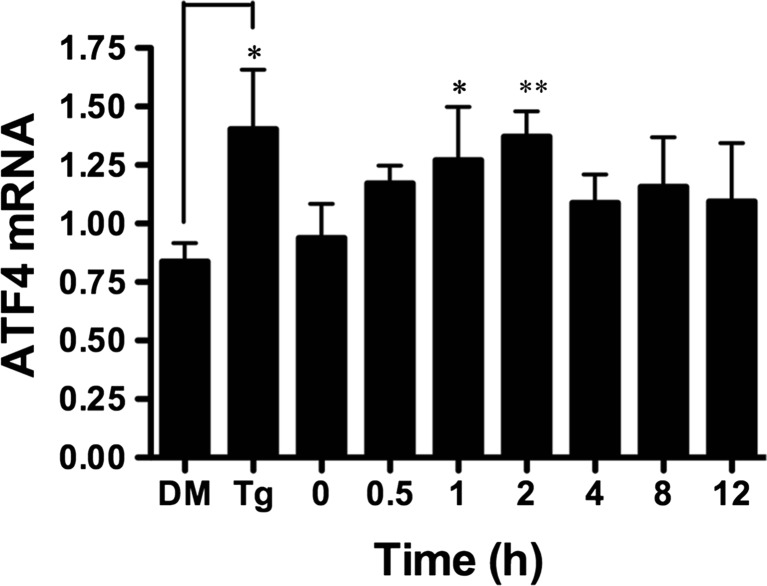
BA induces an increase in ATF4 transcription in DU-145 cells. Ten micromoles of BA induced a significant increase in ATF4 mRNA 1 (*p* = 0.031, *n* = 4) and 2 h (*p* = 0.005, *n* = 4) post-treatment. One micromole of thapsigargin (Tg) and DMSO vehicle (DM) was used as a positive control and significantly induced ATF4 mRNA, *p* < 0.05 (*n* = 5). The gel shown is a representative replicate. A description of the statistical analysis is given in the legend of Fig. 2

**Fig. 6:**
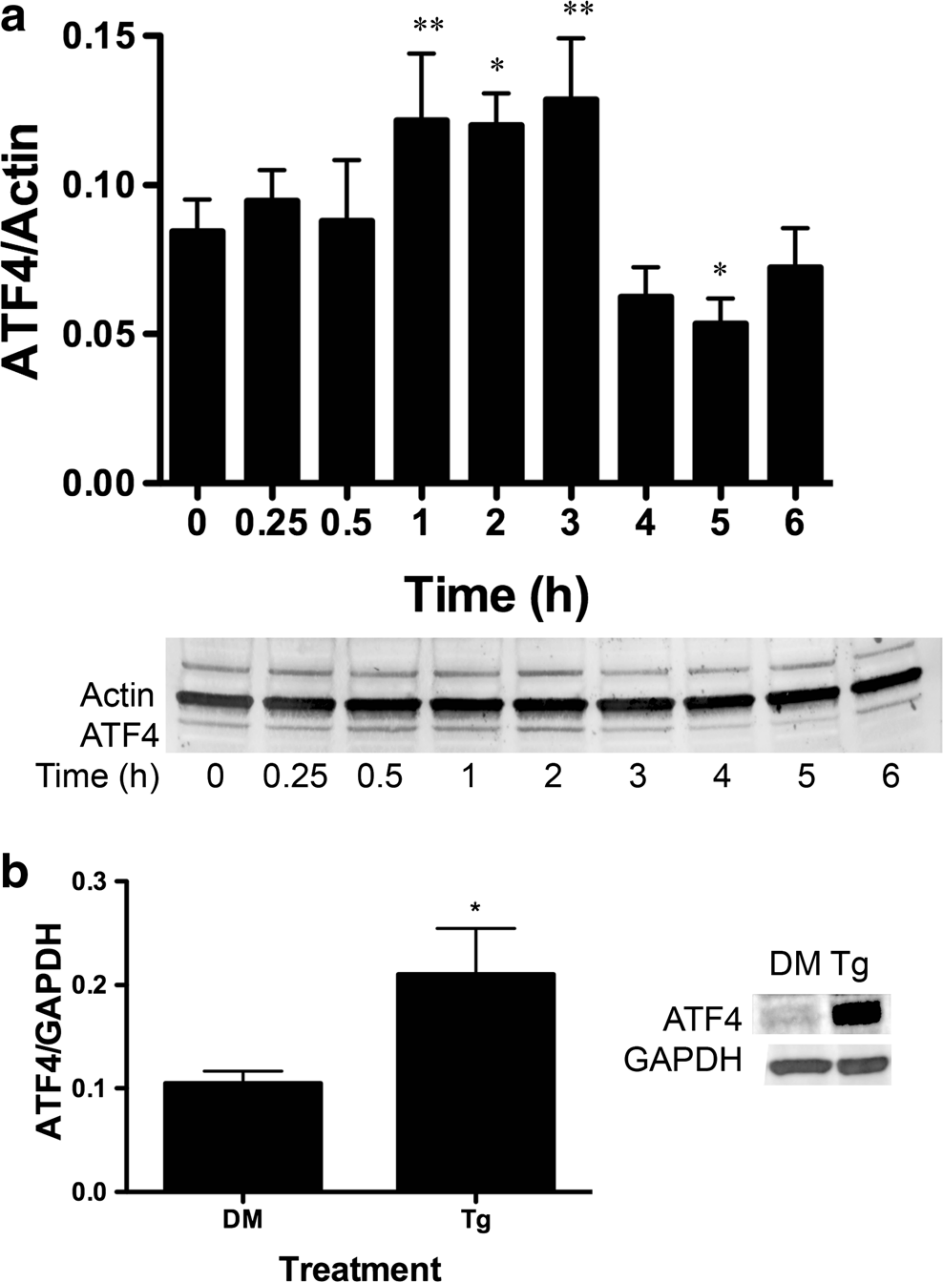
BA induces an increase in ATF4 protein in DU-145 cells. **a** DU-145 cells were treated with 10 μM BA for 0, 0.25, 0.5, 1, 2, 3, 4, 5, and 6 h. ATF4 protein was significantly increased in cells treated at 1 (*p* = 0.01, *n* = 4), 2 (*p* < 0.05, *n* = 3), and 3 h (*p* = 0.004, *n* = 3) and decreased at 5 h (*p* < 0.030, *n* = 4) post-treatment. **b** ATF3 protein was significantly increased in DU-145 cells treated with 1 μM thapsigargin (Tg) or DMSO (DM) for 1 h, (*p* < 0.05 *n* = 3). The gel shown is a representative replicate. A description of the statistical analysis is given in the legend of Fig. 2

Phosphorylation of eIF2α selectively activates transcription and translation of ATF4, a transcription factor in the highly conserved integrated stress response (ISR) that enables cells to survive in adverse environmental conditions. We used RT-PCR to measure ATF4 transcription in DU-145 cells treated with 10 μM BA. ATF4 mRNA was increased at 1 and 2 h post-treatment (Fig. 5). The maximum increase in mRNA was 46 % at 2 h after BA treatment (Table 1). ATF4 mRNA was also elevated in cells treated with thapsigargin (Fig. 5). ATF4 protein was significantly increased from 1 to 3 h followed by a decrease at 5 h post-treatment (Fig. 6a). The maximum increase was 53 % at 3 h (Fig. 6a, Table 1). Thapsigargin treatment also increased ATF4 protein levels (Fig. 6b).

### BA Induces GADD34 and Herp but Decreases CHOP Transcription

**Fig. 7:**
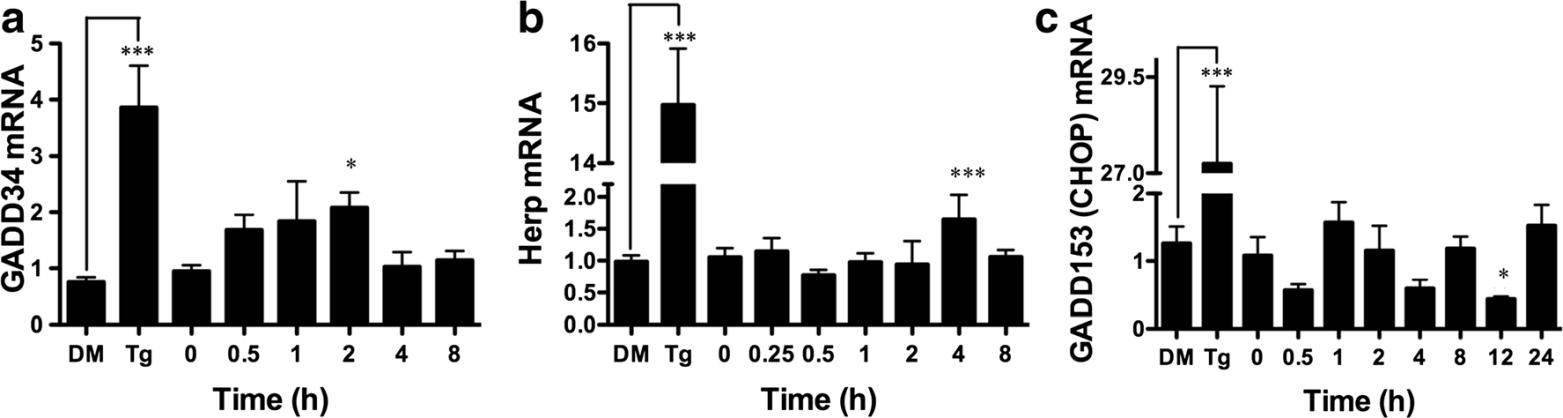
ATF4-inducible genes GADD34 and Herp are upregulated and CHOP downregulated by BA in DU-145 cells. Ten micromoles of BA induced expression of **a** GADD34 at 2 h (*p* < 0.05), *n* = 4) and **b** Herp (*p* < 0.001, *n* = 8)) at 4 h post-treatment and **c** downregulated expression of GADD153 (CHOP) 12 h post-treatment (*p* = 0.012, *n* = 4). As a positive control, 1 μM thapsigargin (Tg) upregulated expression of all three genes compared to DMSO vehicle (DM), (*p* < 0.05, *n* = 3). A description of the statistical analysis is given in the legend of Fig. 2

ATF4 moves into the nucleus where it activates genes that either assist in cell survival by relieving ER stress or promote apoptosis in the case of lethal stresses [36]. GADD34 and homocysteine-induced ER protein (Herp) are ATF4-inducible survival genes, and CHOP (GADD153), which is also induced by ATF4, is a pro-apoptotic gene. We treated DU-145 cells with 10 μM BA and used RT-PCR to analyze changes in mRNA levels. GADD34, which provides a negative feedback loop in the eIF2α/ATF4 pathway by dephosphorylating ph-eIF2α, was significantly increased by 53 % 2 h after treatment (Fig. 7a, Table 1) [36]. Herp is a protein involved in ER-associated degradation (ERAD) that recruits the 26S proteasome component to the ER membrane during ER stress [36]. Herp mRNA was increased by 54 % 4 h after treatment (Fig. 7b, Table 1). In contrast, 10 μM BA caused GADD153/CHOP mRNA expression to decrease. The maximum decrease in mRNA was −59 % at 12 h (Fig. 7c, Table 1).

### BA Decreases CHOP Protein

**Fig. 8:**
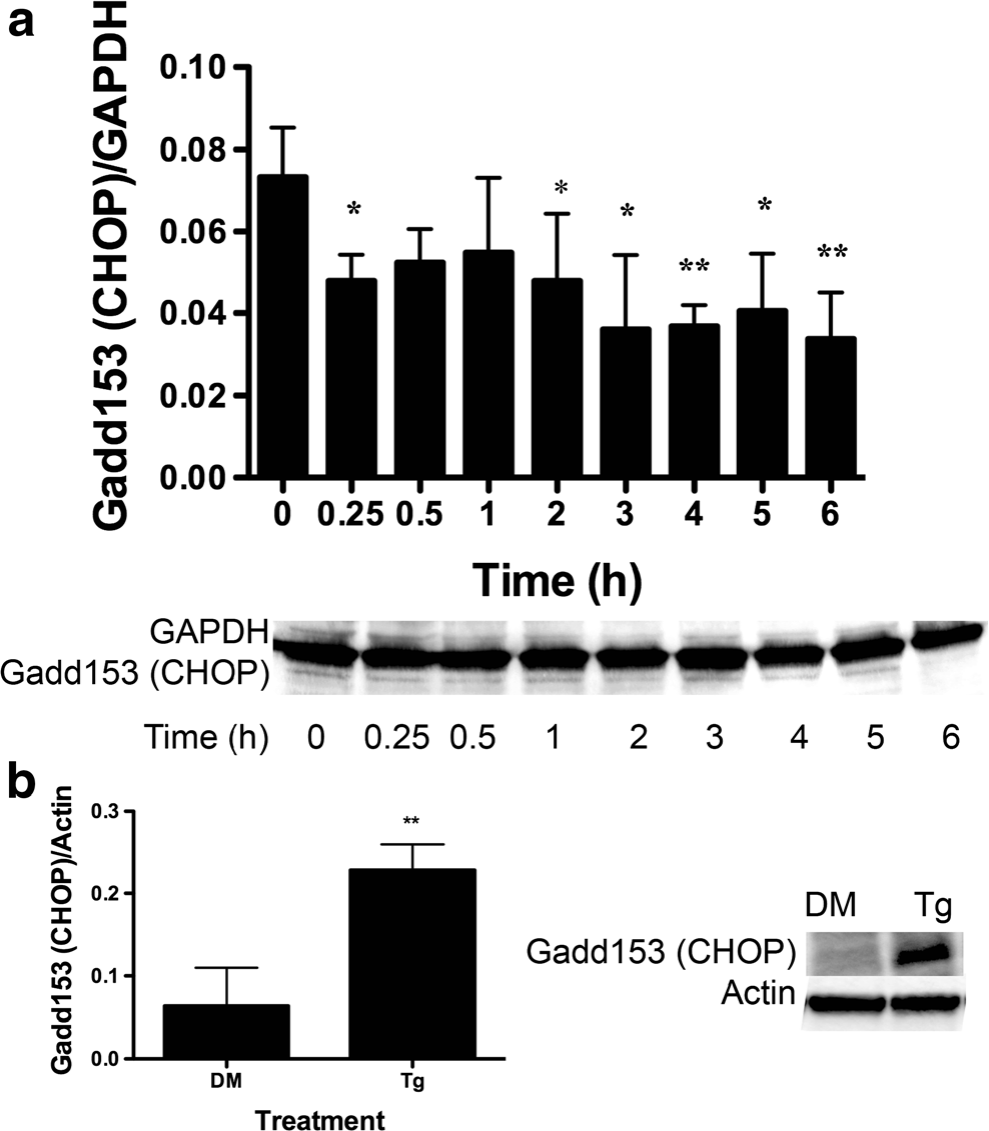
GADD153 (CHOP) protein was reduced in BA-treated DU-145 cells. **a** DU-145 cells treated with 10 μM BA for 0, 0.25, 0.5, 1, 2, 3, 4, 5, and 6 h. GADD153 (CHOP) protein was decreased at 0.25 (*p* < 0.043, *n* = 3), 2 (*p* < 0.033, *n* = 3), 3 (*p* < 0.015, *n* = 4), 4 (*p* < 0.006, *n* = 4), 5 (*p* < 0.014, *n* = 4), and 6 h (*p* < 0.003, *n* = 4). **b** DU-145 cells treated with 1 μM thapsigargin (Tg) or DMSO (DM) vehicle for 1 h. Thapsigargin increased GADD153 (CHOP) protein, (*p* < 0.05, *n* = 3). The gel shown is a representative replicate. A description of the statistical analysis is given in the legend of Fig. 2

We performed immunoblots to measure CHOP/GADD153 protein in DU-145 cells treated with 10 μM BA. CHOP protein expression was significantly decreased at 0.25, 2, 3, 4, 5, and 6 h after treatment (Fig. 8a). The maximum decrease in protein was −51 % at 3 h after BA treatment (Table 1). The increase in CHOP protein expression following thapsigargin treatment showed that DU-145 cells responded normally to a known stimulus of apoptosis (Fig. 8b). The BA-induced decrease in CHOP protein expression was consistent with our observation that the same BA dose decreased CHOP mRNA (Fig. 7c) and with previous findings that DU-145 cells do not undergo apoptosis with BA treatment even at a concentration of 1000 μM, a dose known to cause reproductive and developmental toxicity [14].

### BA Induces ATF6 Activation

**Fig. 9:**
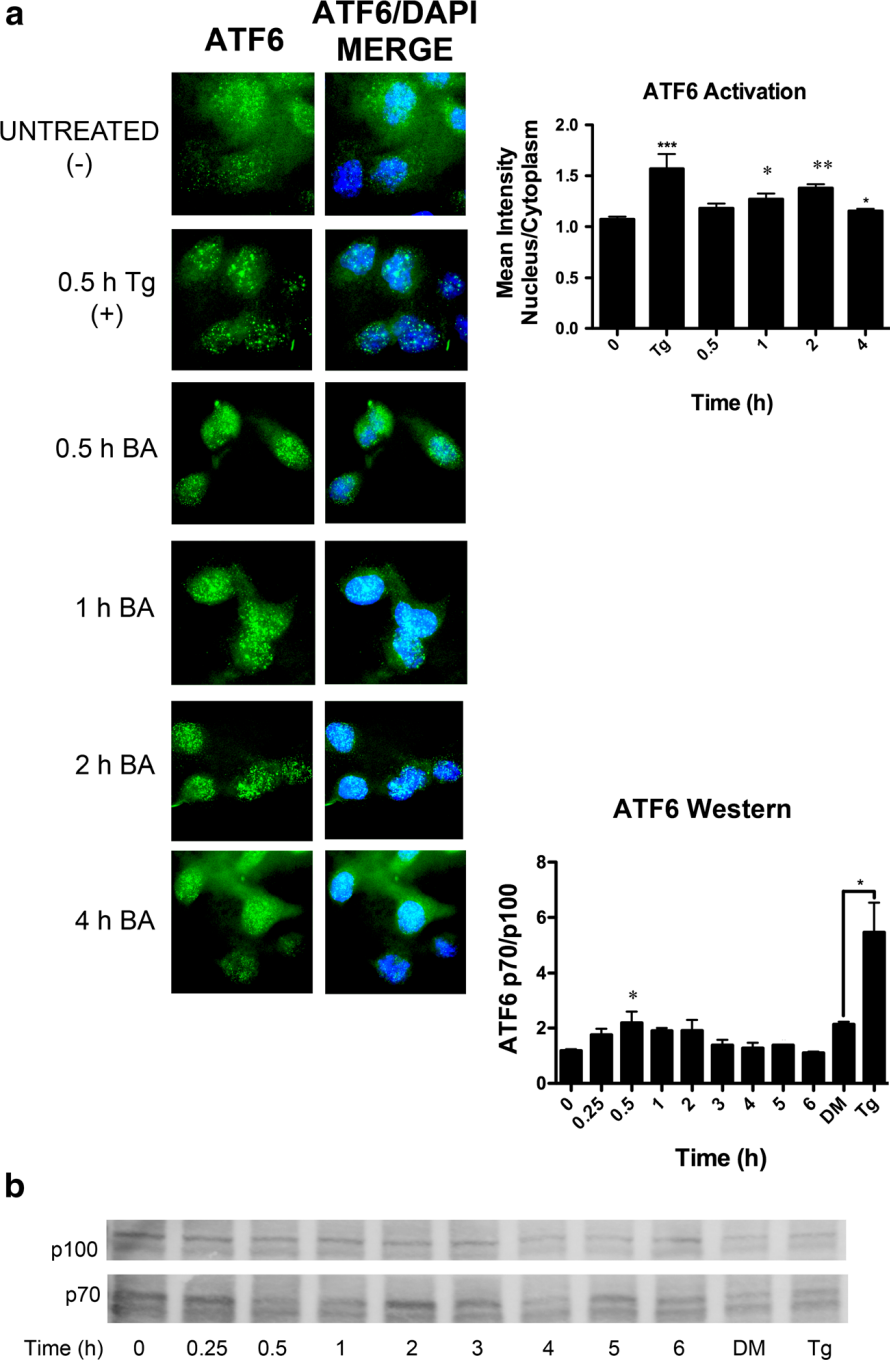
BA stimulates ATF6 activation and translocation to the nucleus. **a** ATF6 (*green*) was present in the nucleus (*blue*) of DU-145 cells 1 (*p* = 0.026, *n* = 17) and 2 h (*p* = 0.002, *n* = 48) post-treatment with 10 μM BA. **b** ATF6 is activated by cleavage. The proportion of the cleaved product (p70) to full length (p100) was significantly elevated 30 min post-treatment (*p* = 0.032, *n* = 3). One micromole of thapsigargin (Tg) also significantly increased the p70/p100 ratio (*p* < 0.05) (*n* = 15). The gel shown is a representative replicate. A description of the statistical analysis is given in the legend of Fig. 2. (Color figure online)

In response to ER stress, the ER resident protein, ATF6, is translocated to the Golgi apparatus where it undergoes proteolysis by the S1P/S2P protease system. The resulting soluble cytoplasmic fragments enter the nucleus and activate transcription of target genes with ER stress response elements (ERSEs), such as GRP78/BiP [37]. We measured the proportion of cleaved ATF6 protein (p70) relative to the full length (p100) and observed an increase 30 min post-treatment (Fig. 9b). ATF6 measured using immunofluorescence was present in the nucleus 1 and 2 h after treatment with 10 μM BA (Fig. 9a). The maximum increase in ATF6 activation was 77 % at 30 min, and the increase in the p70/p100 ratio was 29 % at 2 h (Fig. 9a, b, Table 1).

### BA Increases Transcription of ATF6 Target Genes

**Fig. 10:**
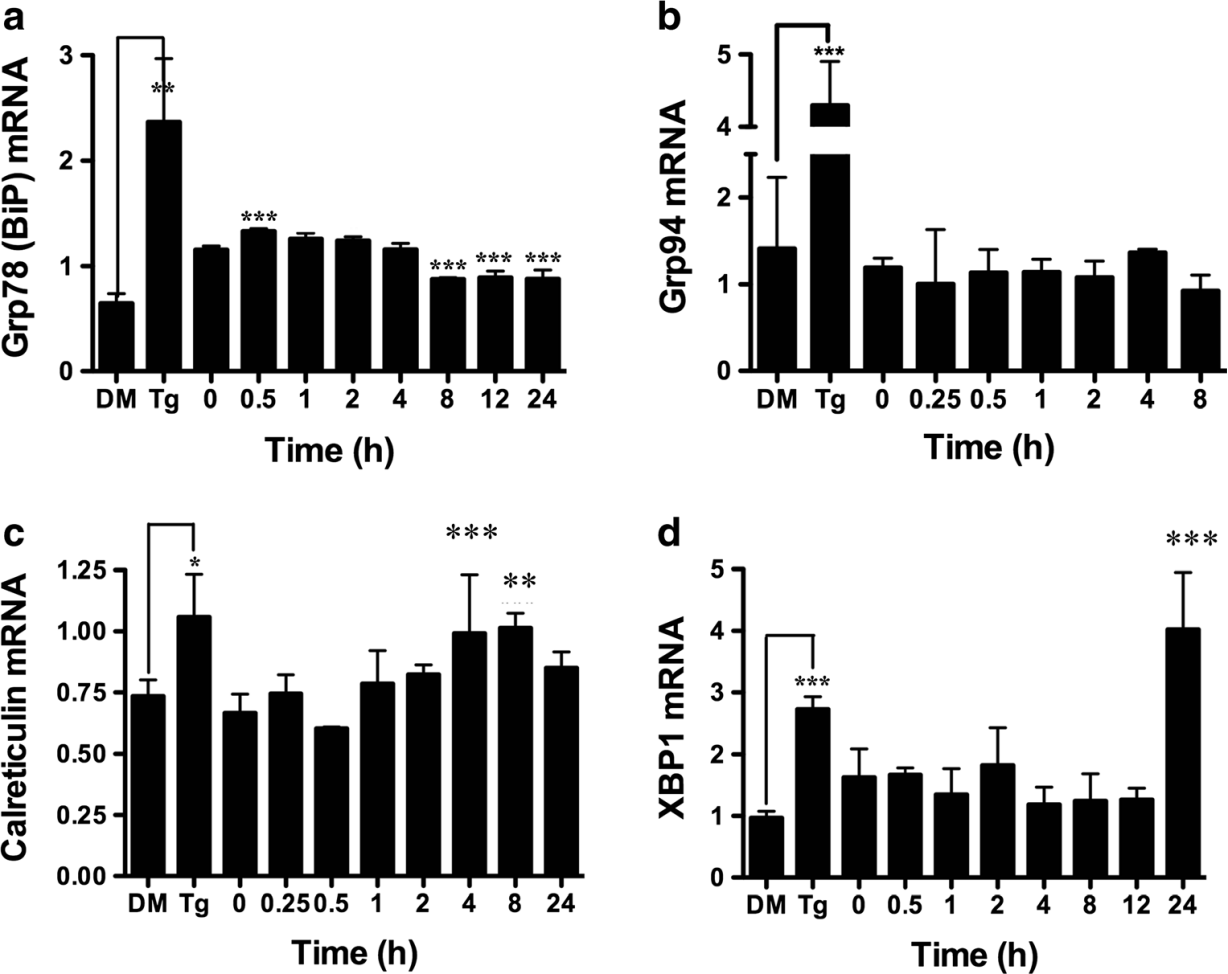
BA upregulates ATF6-inducible genes BiP, GRP94, calreticulin, and XBP1. Ten micromoles of BA induced expression of **a** GRP78 (BiP) at 0.5 h (*p* < 0.001, *n* = 4). GRP78 returned to control levels for several hours and then dropped to lower levels at 8 h (*p* < 0.001, *n* = 4), 12 h (*p* < 0.001, *n* = 4), and 24 h (*p* < 0.001, *n* = 4), suggesting that the cells adapted to higher concentrations of BA. **b** GRP94 was unchanged by treatment. **c** Calreticulin was higher at 4 h (*p* < 0.001, *n* = 5) and 8 h (*p* = 0.003, *n* = 4). **d** XBP1 was higher at 24 h of treatment (*p* < 0.001, *n* = 4). As a positive control, 1 μM thapsigargin (Tg) upregulated expression of all genes compared to DMSO vehicle (DM) (*p* < 0.05, *n* = 4). The gel shown is a representative replicate. A description of the statistical analysis is given in the legend of Fig. 2

Cleaved ATF6 is a transcription factor for genes that contain ERSEI in their promoter [36]. ATF6-inducible genes include the two Ca^2+^-binding proteins GRP78/BiP and calreticulin as well as GRP94 and XBP1 [38]. We measured the mRNA levels of these genes by RT-PCR following treatment of DU-145 cells. Ten-micromole BA treatment increased GRP78/BiP mRNA 30 min after treatment (Fig. 10a) with a maximum of 149 % (Table 1). The maximum increase in BiP/GRP78 protein was 98 % at 1 h (Fig. 4, Table 1). GRP94 was unchanged (Fig. 10b). Calreticulin mRNA increased by 49 % at 4 h (Fig. 10c) with a maximum of 52 % at 8 h after BA treatment (Fig. 10c, Table 1). XBP1 mRNA was increased 24 h post-treatment by 147 % (Fig. 10d, Table 1).

### BA Does Not Activate the IRE1 Branch of UPR

**Fig. 11:**
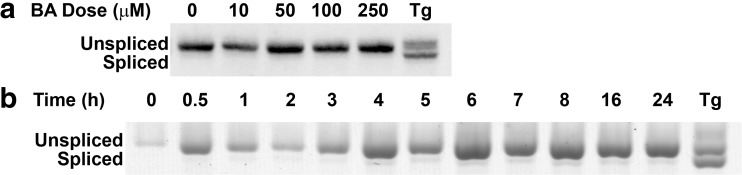
BA does not activate the IRE1 signaling pathway**. a** DU-145 cells were treated with 0, 10, 50, 100, or 250 μM BA or 1 μM thapsigargin (Tg) for 24 h. Thapsigargin was used as a positive control. Increasing BA concentrations for 24 h did not cause XBP1 cleavage. **b** DU-145 cells were treated with 10 μM BA for varying time points or 1 μM thapsigargin (Tg) as a positive control. XBP1 was not cleaved at any time point by BA, but it was by Tg (*p* < 0.05, *n* = 3). The gel shown is a representative replicate

An excessive accumulation of unfolded and misfolded proteins in the ER causes IRE-1 to dimerize allowing *trans*-autophosphorylation of juxtaposed kinase domains and activation of their endoribonuclease domains located in the cytoplasm [39]. Activated IRE1 splices XBP1 mRNA forming pXBP1s (spliced XBP1 protein) that binds to the unfolded protein response element (UPRE) [40]. To assess activation of the IRE1 branch of the UPR, we evaluated splicing of XBP1 mRNA using a primer specific for both spliced and unspliced forms of XBP1 mRNA. When PCR products are run slowly on an agarose gel, the two forms separate into two bands of different mass if IRE-1 is activated and XBP1 mRNA is spliced. We treated DU-145 cells with BA concentrations from 10 to 250 μM for 24 h, and no splicing was observed (Fig. 11a). We also treated DU-145 cells with 10 μM BA for varying time points and again did not observe spliced mRNA (Fig. 11b). These results show that BA does not induce the IRE1 branch of the UPR. Splicing was observed when cells were treated with thapsigargin, a known activator of IRE1 (Fig. 11a, b).

### BA Does Not Activate Hrd1, a Spliced XBP1-Specific Target Gene

**Fig. 12:**
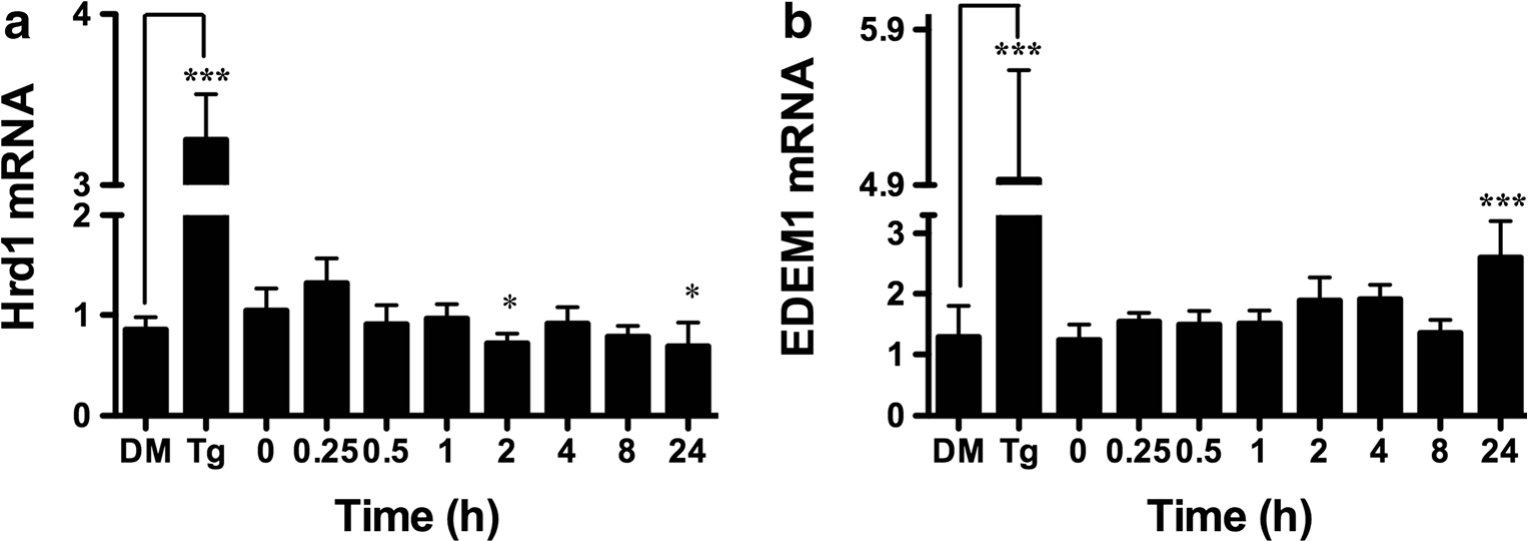
BA does not activate the XBP1s-inducible gene Hrd1, but did activate Edem1 which is also induced by ATF6 and XBP1. **a** Ten micromoles of BA did not induce expression of Hrd1 mRNA in DU-145 cell which dropped below pre-treatment values at 2 h (*p* < 0.05, *n* = 4) and 24 h (*p* < 0.05, *n* = 3). **b** Ten micromoles of BA induced expression of Edem1 at 24 h (*p* < 0.001, *n* = 4) which is regulated by ATF6 and IRE1. As a positive control 1 μM thapsigargin (Tg) upregulated expression of both genes compared to DMSO vehicle (DM), (*n* = 6). The gel shown is a representative replicate. A description of the statistical analysis is given in the legend of Fig. 2

Spliced XBP1 that results from activation of the IRE1 branch acts as a transcription factor for a number of UPR-related genes. If BA activated IRE1, we would expect an increase in the transcription of these genes. To confirm that BA did not activate the IRE1 branch of the UPR, we used RT-PCR to analyze the expression of Hrd1 and ER degradation enhancer mannosidase alpha-like 1 (EDEM1). BA did not increase the transcription of Hrd1 which is specifically activated by spliced XBP1, but not ATF6 (Fig. 12a). EDEM1 is activated by both ATF6 and XBP1 [40]. EDEM1 mRNA was increased at 24 h by treatment with 10 μM BA (Fig. 12b) probably due to activation by ATF6.

### Genes Were Active in the DU-145 Cell Line

The positive control, thapsigargin, demonstrated that all genes evaluated were active in the DU-145 cell line. The Catalogue of Somatic Mutations in Cancer (COSMIC) recorded a mutation in ATF4 in 16.67 % of prostate tumor tissue samples [28]. In the DU-145 cell line, substitution mutations were reported in ATF4 and IRE1 with no phenotypic changes. The mutations did not occur in the regulatory build, and no association was found for any gene measured and copy number or expression in the COSMIC Cell Line. Information available in COSMIC (releases v75 and v76) and Ensemble for each of the genes measured in this manuscript are available in Supplement 2 [28, 41]. ATCC states that DU-145 “is a hypotriploid human cell line. Both 61 and 62 chromosome numbers had the highest rate of occurrence in 30 metaphase counts. The rate of higher ploides was 3 %. The t(11q12q), del(11)q23), 16q+, del(9)(p11),del(1)(p32) and 6 other marker chromosomes were found in most cells. The N13 was usually absent. The Y chromosome is abnormal through translocation to an unidentified chromosomal segment. The X chromosome was present in single copy.”

## Discussion

We describe in this paper the response of DU-145 human prostate cells to concentrations of BA that occur from dietary exposure. We draw seven main conclusions from our results. First, BA reduces the polysome/monosome ratio, an indicator of global protein translation. Changes to the extracellular environment can alter intracellular conditions that stress the ER and inhibit global protein synthesis. Documented stresses include low ER luminal [Ca^2+^]; nutrient stress from glucose deprivation and amino acid deficiency; and toxic exposures from chemical, physical, and biological hazards (NaN_3_, arsenite, oxidants, heat, UV radiation, and viruses) [42–44]. BA treatment lowers luminal ER [Ca^2+^] as does thapsigargin, a plant sesquiterpene lactone, used widely as a strong positive control to assure that cells under study respond to a known ER stressor [15]. ER stress elicits inhibition in global translation by phosphorylation of eIF-2α which is a component of the heterotrimeric eIF-2 complex that loads the initiator transfer RNA (tRNA) (Met-tRNAMet) onto the 40S ribosomal subunit [45–48]. The eIF-2 complex binds GTP/GDP, and its activity is regulated by guanine nucleotide exchange factor eIF-2B. Phosphorylation of eIF-2α increases the affinity of eIF-2 for eIF-2B, preventing the exchange of GDP for GTP. This prevents the formation of the puromycin-sensitive 80S pre-initiation complex and allows elongating ribosomes to release mRNAs, leading to the disassembly of polysomes into monosomes [48]. Here, we show that BA (10 μM) treatment reduced the polysome/monosome ratio by approximately half, an indication that global protein synthesis was inhibited, but not stopped (Fig. 1). Uluisik, Kaya, and colleagues reported that 50 mM BA completely halted polysome formation and protein synthesis in yeast [21].

Second, BA increases the transcription and translation of BiP/GRP78, a resident ER chaperone, with a long half-life (>48 h) and an indicator of ER stress (Figs. 4 and 10) [36, 49]. BiP/GRP78 binds about 20 % of the ER’s Ca^2+^ and is the master regulator of the UPR [34, 50]. In eukaryotic cells, newly synthesized secretory and transmembrane unfolded polypeptides are transported through translocons into the ER lumen. BiP/GRP78 gates the ER side of the tranlocon [34, 51, 52], recognizes hydrophobic regions of nascent unfolded proteins entering the ER, and assists in their folding and assembly into polypeptides [53, 54]. High [Ca^2+^]_ER_ is required for protein folding, and when it is low, unfolded proteins accumulate. BiP/GRP78 activates UPR pathways via its release from resident ER transmembrane proteins to remove the stress [50].

Third, BA activation of eIF2α through phosphorylation at serine 51 is transient. The interaction between BiP/GRP78 and nascent unfolded proteins is stabilized by luminal ER Ca^2+^, and when Ca^2+^ concentrations fall, BiP/GRP78 is released and interacts with PERK, a kinase that phosphorylates eIF2α [50]. Treatment of cells with 10 μM BA significantly elevated the ratio of phosphorylated eIF2α/total eIF2α at 30 min, and it remained high up to 2 h (Fig. 2, Table 1). The decline in ph-eif2α coincided with the rise in GADD34 mRNA, the phosphatase that removes the phosphate from ph-eIF2α (Fig. 7a). Our observation of a return to normal suggests that the cell has completed a change in transcription to the new 10-μM BA environment by 3 h and that a comparison of polysomes before BA treatment and at 3 h would provide a means to identify BA-dependent changes in protein translation.

Fourth, BA upregulates the transcription factor, ATF4. Phosphorylation of eIF2α increases upstream open reading frame (uORF)-mediated translation of bone-related activating transcription factor 4 (ATF4). The ph-eIF2α/ATF4 pathway is highly conserved from yeast to mammals and has been named the ISR because it is a target of many different types of environmental stresses [55]. Treatment with BA resulted in an increase in ATF4 mRNA and protein levels (Figs. 5 and 6, Table 1). ATF4-inducible gene GADD34 increased immediately whereas the increase in Herp, which is dually regulated by ATF6 and ATF4 [56], was delayed to 4 h (Fig. 7b). In neurons, moderate levels of ER stress increase Herp levels, which promotes cell survival by stabilizing ER Ca^2+^, preserving mitochondrial function and suppressing caspase 3, whereas lethal stress levels decrease Herp levels and induce apoptosis [57]. In neuronal PC12 cells, promotion of cell survival is accomplished by Herp’s association with ryanodine and inositol Ca^2+^ channels and facilitation of their proteasome-mediated protein degradation [58].

Fifth, BA reduced the CHOP, also referred to as growth arrest-inducible and DNA damage-inducible gene 153 (GADD153), at both the mRNA and protein levels (Figs. 7c and 8). This outcome is consistent with previous reports showing that BA does not cause apoptosis in DU-145 prostate cells or MDA-MB-231 breast cancer cells at concentrations of 1 mM or less [5, 59]. The absence of CHOP activation by BA is also consistent with the anti-apoptotic activity of BiP/GRP78 and Herp and the absence of BA activation of IRE1 (Figs. 11 and 12a). This can be explained by the low level of eIF2α phosphorylation induced by BA. A single uORF located in the 5′-leader of the CHOP mRNA is responsible for CHOP translation. In non-stress conditions and when eIF2α phosphorylation is low, the uORF serves as a barrier that prevents translation of the downstream CHOP coding region. Lethal stress levels, such as those induced by thapsigargin, induce a high level of eIF2α phosphorylation which facilitates ribosome bypass of the uORF and allows translation [60].

Sixth, BA activates the ATF6 pathway. BA causes a dose- and time-dependent expansion of the ER with the formation of cytoplasmic stress granules (SGs) [19]. ER expansion is induced by ATF6α [61] and independently by pXBP1s via the IRE1 pathway [62, 63]. We show here that treatment with BA activated ATF6α, but not IRE1 (Figs. 9, 10, 11, and 12). ATF6 is retained in the ER bound to BiP/GRP78 and dissociates when unfolded proteins accumulate. ATF6 moves to the Golgi where it is cleaved by S1P and S2P proteases to form a soluble basic leucine zipper (bZIP) transcription factor that moves into the nucleus [34]. Nuclear ATF6 binds to an ERSE promoter located upstream of target genes which include BiP/GRP78, calreticulin, and XBP1 [40]. Calreticulin mRNA increased by 49 % at 4 h (Fig. 10c). Calreticulin is a major ER Ca^2+^-binding protein and increases luminal Ca^2+^ stores [64, 65]. As a chaperone, it recognizes the terminal glucose and four internal mannoses in newly synthesized glycoproteins [66]. Reductions in ER Ca^2+^ decrease the formation of these complexes and ER folding capacity. Calreticulin facilitates the folding of major histocompatibility complex (MHC) class I molecules and their assembly factor tapasin, thereby influencing antigen presentation to cytotoxic T cells [67, 68]. It is also required for the stability and nuclear localization of the p53 protein [69]. Further studies are needed to determine if the ability of BA to induce calreticulin transcription is related to its effects on the immune system [11].

The second ATF6-regulated gene we evaluated was BiP/GRP78. In HeLa cells, thapsigargin induced rapid BiP translation that preceded transcription [70]. The authors suggested that storage of BiP/GRP78 mRNA transcripts allows cells to rapidly synthesize the protein to adapt to small perturbations and reserve transcriptional upregulation for conditions that cause major reductions in the protein level [70].

ATF6 induces XBP1 mRNA under mild stress whereas higher levels of stress induce XBP1 and IRE1-dependent XBP1(s) [40]. ATF6α forms a heterodimer with XBP1 which induces ER degradation enhancing α-mannosidase-like 1 (EDEM1). EDEM1 enhances the release of terminally misfolded glycoproteins from the calnexin chaperone system. In our study, XBP1 and EDEM1 mRNA were increased by 147 and 110 %, respectively, at the 24-h BA treatment (Fig. 12b, Table 1).

Seventh, BA does not activate IRE1. The IRE1 pathway transactivates the transcription of genes encoding ER chaperones and components required for ERAD [71]. High levels of stress induce XBP1 and XBP1(s) which is translated into the XBP1 transcription factor protein [pXBP1(s)] that binds to ERSE and UPRE DNA-binding sites to induce expression of ER chaperones [40]. IRE1 is required for the site-specific cleavage of XBP1 mRNA into fragments that are subsequently ligated to form the transcript encoding pXBP1(s). We did not observe XBP1 cleavage in BA-treated cells at various doses and time points (Fig. 11) nor was there an increase in transcription of Hrd1, a pXBP1(s)-activated gene (Fig. 12a).

In previous studies using DU-145 and LNCaP prostate cancer cells, we showed that long-term exposure to BA (8 days) induces a senescent-like state and inhibits proliferation in a dose-dependent manner [5]. Long-term exposure to high concentrations of BA (250 and 1000 μM) inhibited both cell migration and invasion [72]. Bradke and colleagues extended these observations to shorter periods of exposure and showed that a 24-h exposure to 1000 μM BA inhibited migration of DU-145 cells on fibronectin [73]. The concentrations used in these migration studies are above what human cells would be exposed to from normal dietary intake and occupational exposure [13]. Dose response studies are needed in migration and other studies to clarify those responses that can be achieved by dietary B intake and those that require pharmaceuticals. The observations presented here suggest caution in interpreting genetic responses to BA since they are time dependent. Understandably, this makes the results of single time-point studies using arrays of multiple genes particularly challenging to interpret. It also seems prudent that BA-sensitive periods be identified prior to conducting dose response experiments.

## Conclusion

Dietary boron has been connected to three seemingly unconnected observations, increased bone mass and strength [10, 74, 75], chemoprevention [1, 3, 4, 6], and prevention of retinal degeneration [76]. BiP/GRP78 is expressed in the mineralizing matrices of teeth and bone and in the extracellular matrix of differentiating human marrow stromal cells and dental pulp stem cells. BiP/GRP78 binds to type I collagen and dentin matrix protein 1 and aids in the nucleation of calcium phosphate [77]. ATF4 regulates osteogenesis during development and postnatal bone remodeling and upregulates osteocalcin [78]. Parathyroid hormone, an essential regulator of endochondral bone formation and an important anabolic agent for the reversal of bone loss, mediates its functions in part by regulating the binding of ATF4 to the osteoblast-specific gene, osteocalcin [79]. BiP/GRP78 and EDEM prevent aggregation of misfolded opsin leading to retinal degeneration [80, 81]. Our present observation that BA increased both BiP/GRP78 and EDEM provides a hypothesis for the observed retinal degeneration in B-depleted zebrafish [76].

BA inhibits the cADPR Ca^2+^ pathway which activates cell proliferation and inhibits differentiation [15, 82] and activates TIA-1 [18] which has been reported to inhibit tumor progression and invasion [83]. Here, we show that BA treatment increased GRP78 which inhibits cell migration [84] and calreticulin which suppresses prostate cancer by inhibiting growth and metastasis [85]. Calreticulin is also necessary for p53 function an important tumor suppressor [69].The eIF2α/ATF4 and ATF6 pathways are downregulated in genetic models of prostate cancer, and their activation by BA is consistent with its reported chemopreventative effect in human populations [86].

Our studies were performed at a BA concentration that can be achieved by diet. The median consumption of boron in the USA ranges from 0.75 to 1.35 mg B/day in adults and is derived primarily from dietary plants. Borates provide the essential diester link between chains of the pectic polysaccharide rhamnogalactanuran II (RG-II) that is essential for plant growth. RGII occurs in the cell walls of gymnosperms and angiosperms and is a soluble plant fiber that constitutes 15–30 % of dietary fiber [87]. Boron-rich food sources include tree nuts and peanuts, fruits, vegetables, legumes, and wine which, along with olive oil, are the signature of the Mediterranean diet. The NIH-AARP study reported that this diet decreased cancer mortality by 17 % in men and 13 % in women during a 5-year study period [88]. Determining how important boron was to this outcome will require teasing out the relative contributions of dietary components. In the meantime, we find it of interest that coffee in North America and soybeans in China are the major contributors of boron to the diet [89, 90], and both have been associated with reduced risk of prostate cancer [91, 92].

## Electronic supplementary material

ESM 1 (DOCX 49 kb)

ESM 2 (DOCX 19 kb)

## Abbreviations

*BA*: Boric acid
*cADPR*: Cyclic ADP ribose
*ER*: Endoplasmic reticulum
*UPR*: Unfolded protein response
*GADD34*: Growth arrest and DNA damage-inducible protein 34
HERP: Homocysteine-responsive endoplasmic reticulum resident ubiquitin-like domain member 1 protein
CHOP: CCAAT/enhancer-binding protein homologous protein
GRP78: Binding immunoglobulin protein (BiP) (also known as 78-kDa glucose-regulated protein)
GRP94: 94-kDa glucose-regulated (also known as protein heat shock protein 90-kDa beta member 1)
XBP-1: Xbox binding protein
EDEM1: ER degradation enhancer mannosidase alpha-like 1
mRNP: Ribonuclear protein particles
ATF4: Activating transcription factor 4
ISR: Integrated stress response
ESRE1: Endoplasmic reticulum stress response element 1
