# Supplementary material for: Activation of the EIF2α/ATF4 and ATF6 Pathways in DU-145 Cells by Boric Acid at the Concentration Reported in Men at the US Mean Boron Intake
Source: Biol Trace Elem Res. 2016 Sep 1;176(2):278–93. doi: 10.1007/s12011-016-0824-y (PMC5344959; doi:10.1007/s12011-016-0824-y)
Supplement: Supplementary file 1 — (DOCX 49 kb) [file 12011_2016_824_MOESM1_ESM.docx]

**Supplement 1**

**Figure 1 Polysome Profiles**

**Paired t-test:**

**Data source:** Data 1 in Notebook1 Polysomes

**Normality Test (Shapiro-Wilk):**  Passed (P = 0.608)

**Treatment Name N Missing Mean Std Dev SEM**

0 3 0 5.045 0.115 0.0663

10 3 0 2.326 0.112 0.0645

Difference 3 0 2.720 0.0285 0.0165

t = 165.038 with 2 degrees of freedom.

95 percent two-tailed confidence interval for difference of means: 2.649 to 2.791

Two-tailed P-value = 0.0000367

The change that occurred with the treatment is greater than would be expected by chance; there is a statistically significant change (P = <0.001)

One-tailed P-value = 0.0000184

The sample mean of treatment 0 exceeds the sample mean of treatment 10 by an amount that is greater than would be expected by chance, rejecting the hypothesis that the population mean of treatment 10 is greater than or equal to the population mean of treatment 0. (P = <0.001)

Power of performed two-tailed test with alpha = 0.050: 1.000

Power of performed one-tailed test with alpha = 0.050: 1.000

**Figure 2 ph-eif2/total eif2**

**One Way Repeated Measures Analysis of Variance**

**Data source:** Data 1 in Notebook 1 ph-eif2

**Normality Test (Shapiro-Wilk):**  Passed (P = 0.292)

**Equal Variance Test (Brown-Forsythe):** Passed (P = 0.708)

**Treatment Name N Missing Mean Std Dev SEM**

control 0 h 5 0 0.0551 0.0262 0.0117

0.25 4 0 0.0885 0.0214 0.0107

0.5 3 0 0.111 0.00373 0.00215

1 3 0 0.124 0.0151 0.00872

2 3 0 0.112 0.00934 0.00539

3 3 0 0.0743 0.0207 0.0119

4 4 0 0.0814 0.0191 0.00953

5 5 0 0.0702 0.0144 0.00646

5 5 0 0.0702 0.0144 0.00646

6 4 0 0.0569 0.0229 0.0115

**Source of Variation DF SS MS F P**

Between Subjects 4 0.00293 0.000733

Between Treatments 9 0.0133 0.00148 5.257 <0.001

Residual 25 0.00704 0.000282

Total 38 0.0282 0.000742

The differences in the mean values among the treatment groups are greater than would be expected by chance; there is a statistically significant difference (P = <0.001). To isolate the group or groups that differ from the others use a multiple comparison procedure.

Power of performed test with alpha = 0.050: 0.978

Expected Mean Squares:

Approximate DF Residual = 25.000

Expected MS(Subj) = var(res) + 7.250 var(Subj)

Expected MS(Treatment) = var(res) + var(Treatment)

Expected MS(Residual) = var(res)

Multiple Comparisons versus Control Group (Holm-Sidak method):

Overall significance level = 0.05

Comparisons for factor:

**Comparison Diff of Means t P P<0.050**

control 0 h vs. 1 0.0604 4.796 <0.001 Yes

control 0 h vs. 2 0.0492 3.902 0.005 Yes

control 0 h vs. 0.5 0.0476 3.776 0.006 Yes

control 0 h vs. 0.25 0.0293 2.555 0.098 No

control 0 h vs. 4 0.0222 1.934 0.284 No

control 0 h vs. 5 0.0151 1.427 0.516 No

control 0 h vs. 5 0.0151 1.427 0.420 No

control 0 h vs. 3 0.0111 0.880 0.624 No

control 0 h vs. 6 0.00235 0.205 0.839 No

**Figure 3 GADD34 immunoblot**

**One Way Repeated Measures Analysis of Variance**

**Data source:** Data 1 Notebook 1 GADD34 western.JNB

**Normality Test (Shapiro-Wilk):**  Passed (P = 0.231)

**Equal Variance Test (Brown-Forsythe):** Passed (P = 0.837)

**Treatment Name N Missing Mean Std Dev SEM**

0 3 0 0.0641 0.0177 0.0102

.25 3 0 0.0833 0.0127 0.00735

.5 3 0 0.0829 0.00940 0.00543

1 3 0 0.0877 0.0129 0.00745

2 3 0 0.0956 0.0145 0.00838

3 3 0 0.0979 0.0108 0.00625

4 5 0 0.0697 0.0158 0.00706

5 4 0 0.0629 0.0143 0.00713

6 4 0 0.0565 0.0208 0.0104

**Source of Variation DF SS MS F P**

Between Subjects 4 0.00326 0.000816

Between Treatments 8 0.00533 0.000667 6.802 <0.001

Residual 18 0.00176 0.0000980

Total 30 0.0112 0.000372

The differences in the mean values among the treatment groups are greater than would be expected by chance; there is a statistically significant difference (P = <0.001). To isolate the group or groups that differ from the others use a multiple comparison procedure.

Power of performed test with alpha = 0.050: 0.991

Expected Mean Squares:

Approximate DF Residual = 18.000

Expected MS(Subj) = var(res) + 5.500 var(Subj)

Expected MS(Treatment) = var(res) + var(Treatment)

Expected MS(Residual) = var(res)

Multiple Comparisons versus Control Group (Holm-Sidak method):

Overall significance level = 0.05

Comparisons for factor:

**Comparison Diff of Means t P P<0.050**

0 vs. 3 0.0338 3.769 0.011 Yes

0 vs. 2 0.0315 3.506 0.018 Yes

0 vs. 1 0.0236 2.634 0.097 No

0 vs. .25 0.0192 2.138 0.212 No

0 vs. .5 0.0188 2.091 0.189 No

0 vs. 6 0.00644 0.771 0.834 No

0 vs. 4 0.00355 0.459 0.879 No

0 vs. 5 0.0000128 0.00153 0.999 No

**Figure 4 Grp78 (BiP )immunoblot**

**Data source:** Data 1 in Notebook 1 Grp78 (BiP) immunoblot

**Normality Test (Shapiro-Wilk):**  Passed (P = 0.321)

**Equal Variance Test (Brown-Forsythe):** Passed (P = 0.093)

**Treatment Name N Missing Mean Std Dev SEM**

0 4 0 0.0781 0.0145 0.00725

0.25 4 0 0.119 0.0365 0.0183

0.5 4 0 0.141 0.0306 0.0153

1 4 0 0.155 0.0374 0.0187

2 4 0 0.138 0.0312 0.0156

3 4 0 0.149 0.0178 0.00888

4 4 0 0.126 0.0272 0.0136

5 4 0 0.129 0.0335 0.0168

6 4 0 0.102 0.0384 0.0192

**Source of Variation DF SS MS F P**

Between Subjects 3 0.00611 0.00204

Between Treatments 8 0.0185 0.00231 2.863 0.022

Residual 24 0.0194 0.000808

Total 35 0.0440

The differences in the mean values among the treatment groups are greater than would be expected by chance; there is a statistically significant difference (P = 0.022). To isolate the group or groups that differ from the others use a multiple comparison procedure.

Power of performed test with alpha = 0.050: 0.641

Multiple Comparisons versus Control Group (Holm-Sidak method):

Overall significance level = 0.05

Comparisons for factor:

**Comparison Diff of Means t P P<0.050**

0 vs. 1 0.0766 3.813 0.007 Yes

0 vs. 3 0.0704 3.505 0.013 Yes

0 vs. 0.5 0.0627 3.122 0.028 Yes

0 vs. 2 0.0600 2.987 0.032 Yes

0 vs. 5 0.0508 2.529 0.072 No

0 vs. 4 0.0481 2.391 0.073 No

0 vs. 0.25 0.0407 2.023 0.106 No

0 vs. 6 0.0240 1.194 0.244 No

**Figure 5 ATF4 mRNA**

**One Way Repeated Measures Analysis of Variance**

**Data source:** Data 1 in Notebook 1 ATF4 mRNA. JNB.

**Normality Test (Shapiro-Wilk):**  Passed (P = 0.268)

**Equal Variance Test (Brown-Forsythe):** Passed (P = 0.412)

**Treatment Name N Missing Mean Std Dev SEM**

0 5 0 0.938 0.147 0.0656

.5 4 0 1.172 0.0748 0.0374

1 4 0 1.271 0.227 0.113

2 4 0 1.371 0.109 0.0543

4 5 0 1.088 0.121 0.0542

8 3 0 1.157 0.212 0.123

12 3 0 1.094 0.249 0.144

**Source of Variation DF SS MS F P**

Between Subjects 4 0.140 0.0349

Between Treatments 6 0.478 0.0796 3.181 0.028

Residual 17 0.426 0.0250

Total 27 1.074 0.0398

The differences in the mean values among the treatment groups are greater than would be expected by chance; there is a statistically significant difference (P = 0.028). To isolate the group or groups that differ from the others use a multiple comparison procedure.

Power of performed test with alpha = 0.050: 0.605

Expected Mean Squares:

Approximate DF Residual = 17.000

Expected MS(Subj) = var(res) + 5.250 var(Subj)

Expected MS(Treatment) = var(res) + var(Treatment)

Expected MS(Residual) = var(res)

Multiple Comparisons versus Control Group (Holm-Sidak method):

Overall significance level = 0.05

Comparisons for factor:

**Comparison Diff of Means t P P<0.050**

0 vs. 2 0.439 4.027 0.005 Yes

0 vs. 1 0.339 3.112 0.031 Yes

0 vs. .5 0.240 2.205 0.156 No

0 vs. 8 0.252 2.101 0.145 No

0 vs. 12 0.189 1.578 0.248 No

0 vs. 4 0.150 1.503 0.151 No

**Figure 6 ATF4 immunoblot**

**One Way Repeated Measures Analysis of Variance**

**Data source:** Data 1 in Notebook 1 ATF4 immunoblot .JNB

**Normality Test (Shapiro-Wilk):**  Passed (P = 0.328)

**Equal Variance Test (Brown-Forsythe):** Passed (P = 0.546)

**Treatment Name N Missing Mean Std Dev SEM**

0 4 0 0.0844 0.0109 0.00543

.25 5 0 0.0946 0.0104 0.00465

.5 4 0 0.0880 0.0204 0.0102

1 4 0 0.122 0.0224 0.0112

2 3 0 0.120 0.0108 0.00621

3 3 0 0.129 0.0206 0.0119

4 4 0 0.0624 0.00996 0.00498

5 4 0 0.0534 0.00846 0.00423

6 5 0 0.0723 0.0133 0.00593

**Source of Variation DF SS MS F P**

Between Subjects 4 0.00145 0.000363

Between Treatments 8 0.0212 0.00265 13.898 <0.001

Residual 23 0.00439 0.000191

Total 35 0.0272 0.000776

The differences in the mean values among the treatment groups are greater than would be expected by chance; there is a statistically significant difference (P = <0.001). To isolate the group or groups that differ from the others use a multiple comparison procedure.

Power of performed test with alpha = 0.050: 1.000

Expected Mean Squares:

Approximate DF Residual = 23.000

Expected MS(Subj) = var(res) + 6.750 var(Subj)

Expected MS(Treatment) = var(res) + var(Treatment)

Expected MS(Residual) = var(res)

Multiple Comparisons versus Control Group (Holm-Sidak method):

Overall significance level = 0.05

Comparisons for factor:

**Comparison Diff of Means t P P<0.050**

0 vs. 3 0.0454 4.088 0.004 Yes

0 vs. 1 0.0372 3.634 0.010 Yes

0 vs. 2 0.0367 3.310 0.018 Yes

0 vs. 5 0.0310 3.022 0.030 Yes

0 vs. 4 0.0220 2.144 0.161 No

0 vs. 6 0.0117 1.226 0.548 No

0 vs. .25 0.0106 1.117 0.475 No

0 vs. .5 0.00360 0.352 0.728 No

**Figure 7A GADD34 mRNA**

**Kruskal-Wallis One Way Analysis of Variance on Ranks**

**Data source:** Data 1 in Notebook 1 GADD34 mRNA1.JNB

**Normality Test (Shapiro-Wilk):**  Passed (P = 0.946)

**Equal Variance Test (Brown-Forsythe):** Failed (P < 0.050)

**Group N Missing Median 25% 75%**

0 4 0 0.933 0.853 1.058

.5 4 0 1.689 1.428 1.943

1 4 0 1.835 1.204 2.484

2 4 0 2.100 1.820 2.322

4 4 0 0.918 0.863 1.298

8 5 0 1.147 0.995 1.286

H = 16.693 with 5 degrees of freedom. (P = 0.005)

The differences in the median values among the treatment groups are greater than would be expected by chance; there is a statistically significant difference (P = 0.005)

To isolate the group or groups that differ from the others use a multiple comparison procedure.

Multiple Comparisons versus Control Group (Dunn's Method) :

**Comparison Diff of Ranks Q P P<0.050**

2 vs 0 15.750 3.026 0.012 Yes

1 vs 0 13.000 2.498 0.062 No

.5 vs 0 12.500 2.402 0.082 Do Not Test

8 vs 0 4.550 0.922 1.000 Do Not Test

4 vs 0 1.500 0.288 1.000 Do Not Test

**Figure 7B Herp mRNA**

**One Way Repeated Measures Analysis of Variance**

**Data source:** Data 1 in Notebook1 Herp mRNA

**Normality Test (Shapiro-Wilk):**  Passed (P = 0.618)

**Equal Variance Test (Brown-Forsythe):** Passed (P = 0.122)

**Treatment Name N Missing Mean Std Dev SEM**

0 8 0 1.055 0.140 0.0496

.25 9 0 1.146 0.210 0.0700

.5 8 2 0.774 0.0824 0.0336

1 9 0 0.977 0.138 0.0460

2 8 0 0.940 0.366 0.129

4 8 2 1.648 0.383 0.156

8 8 0 1.056 0.110 0.0388

**Source of Variation DF SS MS F P**

Between Subjects 8 0.451 0.0564

Between Treatments 6 2.399 0.400 7.874 <0.001

Residual 39 1.980 0.0508

Total 53 5.230 0.0987

The differences in the mean values among the treatment groups are greater than would be expected by chance; there is a statistically significant difference (P = <0.001). To isolate the group or groups that differ from the others use a multiple comparison procedure.

Power of performed test with alpha = 0.050: 0.998

Expected Mean Squares:

Approximate DF Residual = 39.000

Expected MS(Subj) = var(res) + 5.875 var(Subj)

Expected MS(Treatment) = var(res) + var(Treatment)

Expected MS(Residual) = var(res)

Multiple Comparisons versus Control Group (Holm-Sidak method):

Overall significance level = 0.05

Comparisons for factor:

**Comparison Diff of Means t P P<0.050**

0 vs. 4 0.567 4.497 <0.001 Yes

0 vs. .5 0.252 2.001 0.236 No

0 vs. 2 0.115 0.991 0.796 No

0 vs. .25 0.102 0.920 0.742 No

0 vs. 1 0.0669 0.602 0.798 No

0 vs. 8 0.00149 0.0129 0.990 No

**Figure 7C GADD153 (CHOP) mRNA**

**One Way Repeated Measures Analysis of Variance**

**Data source:** Data 1 in Notebook 1 GADD153 (chop) mRNA.JNB

**Normality Test (Shapiro-Wilk):**  Passed (P = 0.489)

**Equal Variance Test (Brown-Forsythe):** Passed (P = 0.629)

**Treatment Name N Missing Mean Std Dev SEM**

0 4 0 1.081 0.275 0.137

.5 4 0 0.567 0.0911 0.0456

1 3 0 1.570 0.302 0.174

2 5 0 1.156 0.365 0.163

4 4 0 0.595 0.132 0.0659

8 4 0 1.183 0.177 0.0884

12 4 0 0.440 0.0355 0.0177

24 3 0 1.525 0.305 0.176

**Source of Variation DF SS MS F P**

Between Subjects 4 0.318 0.0795

Between Treatments 7 4.852 0.693 13.392 <0.001

Residual 19 0.983 0.0518

Total 30 6.042 0.201

The differences in the mean values among the treatment groups are greater than would be expected by chance; there is a statistically significant difference (P = <0.001). To isolate the group or groups that differ from the others use a multiple comparison procedure.

Power of performed test with alpha = 0.050: 1.000

Expected Mean Squares:

Approximate DF Residual = 19.000

Expected MS(Subj) = var(res) + 5.750 var(Subj)

Expected MS(Treatment) = var(res) + var(Treatment)

Expected MS(Residual) = var(res)

Multiple Comparisons versus Control Group (Holm-Sidak method):

Overall significance level = 0.05

Comparisons for factor:

**Comparison Diff of Means t P P<0.050**

0 vs. 12 0.641 3.636 0.012 Yes

0 vs. .5 0.514 2.916 0.052 No

0 vs. 4 0.486 2.756 0.061 No

0 vs. 1 0.512 2.693 0.056 No

0 vs. 24 0.466 2.452 0.070 No

0 vs. 2 0.162 1.009 0.545 No

0 vs. 8 0.103 0.584 0.566 No

**Figure 8 GADD153 (CHOP) immunoblot**

**One Way Repeated Measures Analysis of Variance**

**Data source:** Data 1 in Notebook1 GADD153 (chop) immunoblot

**Normality Test (Shapiro-Wilk):**  Passed (P = 0.641)

**Equal Variance Test (Brown-Forsythe):** Passed (P = 0.728)

**Treatment Name N Missing Mean Std Dev SEM**

0 3 0 0.0733 0.0121 0.00697

0.25 3 0 0.0479 0.00645 0.00372

.5 4 0 0.0525 0.00811 0.00405

1 4 0 0.0548 0.0183 0.00914

2 3 0 0.0479 0.0165 0.00951

3 4 1 0.0360 0.0183 0.0105

4 4 0 0.0368 0.00522 0.00261

5 4 0 0.0406 0.0140 0.00702

6 4 0 0.0337 0.0114 0.00568

**Source of Variation DF SS MS F P**

Between Subjects 3 0.00148 0.000494

Between Treatments 8 0.00332 0.000415 3.514 0.011

Residual 20 0.00236 0.000118

Total 31 0.00794 0.000256

The differences in the mean values among the treatment groups are greater than would be expected by chance; there is a statistically significant difference (P = 0.011). To isolate the group or groups that differ from the others use a multiple comparison procedure.

Power of performed test with alpha = 0.050: 0.768

Expected Mean Squares:

Approximate DF Residual = 20.000

Expected MS(Subj) = var(res) + 7.667 var(Subj)

Expected MS(Treatment) = var(res) + var(Treatment)

Expected MS(Residual) = var(res)

Multiple Comparisons versus Control Group (Holm-Sidak method):

Overall significance level = 0.05

Comparisons for factor:

**Comparison Diff of Means t P P<0.050**

0 vs. 6 0.0360 4.286 0.003 Yes

0 vs. 4 0.0329 3.922 0.006 Yes

0 vs. 5 0.0291 3.470 0.014 Yes

0 vs. 3 0.0305 3.374 0.015 Yes

0 vs. 0.25 0.0254 2.802 0.043 Yes

0 vs. 2 0.0254 2.800 0.033 Yes

0 vs. .5 0.0172 2.050 0.104 No

0 vs. 1 0.0149 1.770 0.092 No

**Figure 9A ATF6 activation ATF6 DAPI merge in cells**

**One Way Repeated Measures Analysis of Variance**

**Data source:** Data 1 in Notebook 1 ATF6 activation ATF6 DAPI merge in cell.JNB

**Normality Test (Shapiro-Wilk):**  Failed (P < 0.050)

**Equal Variance Test (Brown-Forsythe):** Passed (P = 0.484)

**Treatment Name N Missing Mean Std Dev SEM**

0 19 0 1.072 0.118 0.0271

0.5 31 0 1.181 0.256 0.0460

1 17 0 1.270 0.233 0.0565

2 48 0 1.380 0.261 0.0376

4 48 0 1.153 0.160 0.0231

**Source of Variation DF SS MS F P**

Between Subjects 47 2.451 0.0522

Between Treatments 4 1.674 0.419 9.230 <0.001

Residual 111 5.033 0.0453

Total 162 9.415 0.0581

The differences in the mean values among the treatment groups are greater than would be expected by chance; there is a statistically significant difference (P = <0.001). To isolate the group or groups that differ from the others use a multiple comparison procedure.

Power of performed test with alpha = 0.050: 0.998

Expected Mean Squares:

Approximate DF Residual = 111.000

Expected MS(Subj) = var(res) + 3.362 var(Subj)

Expected MS(Treatment) = var(res) + var(Treatment)

Expected MS(Residual) = var(res)

Multiple Comparisons versus Control Group (Holm-Sidak method):

Overall significance level = 0.05

Comparisons for factor:

**Comparison Diff of Means t P P<0.050**

0 vs. 2 0.227 3.640 0.002 Yes

0 vs. 1 0.212 2.675 0.026 Yes

0 vs. 0.5 0.0654 0.956 0.566 No

0 vs. 4 0.000978 0.0157 0.988 No

**Figure 9B ATF6 p70/p100**

**One Way Repeated Measures Analysis of Variance**

**Data source:** Data 1 in Notebook 1 ATF7 p70 cleaved product divided by p100 full length.JNB

**Normality Test (Shapiro-Wilk):**  Passed (P = 0.070)

**Equal Variance Test (Brown-Forsythe):** Passed (P = 0.950)

**Treatment Name N Missing Mean Std Dev SEM**

0 3 0 1.184 0.0861 0.0497

.25 3 0 1.758 0.382 0.221

.5 3 0 2.187 0.714 0.412

1 3 0 1.906 0.171 0.0988

2 3 0 1.909 0.676 0.390

2 3 0 1.909 0.676 0.390

3 3 0 1.381 0.333 0.192

4 3 0 1.279 0.335 0.193

5 3 0 1.381 0.0353 0.0204

6 3 0 1.101 0.0873 0.0504

**Source of Variation DF SS MS F P**

Between Subjects 2 1.244 0.622

Between Treatments 9 3.828 0.425 3.145 0.018

Residual 18 2.434 0.135

Total 29 7.505

The differences in the mean values among the treatment groups are greater than would be expected by chance; there is a statistically significant difference (P = 0.018). To isolate the group or groups that differ from the others use a multiple comparison procedure.

Power of performed test with alpha = 0.050: 0.693

Multiple Comparisons versus Control Group (Holm-Sidak method):

Overall significance level = 0.05

Comparisons for factor:

**Comparison Diff of Means t P P<0.050**

0 vs. .5 1.003 3.342 0.032 Yes

0 vs. 2 0.726 2.417 0.193 No

0 vs. 2 0.726 2.417 0.171 No

0 vs. 1 0.722 2.405 0.152 No

0 vs. .25 0.575 1.914 0.310 No

0 vs. 5 0.198 0.659 0.946 No

0 vs. 3 0.198 0.658 0.888 No

0 vs. 4 0.0950 0.317 0.940 No

0 vs. 6 0.0829 0.276 0.786 No

**Figure 10A Grp78 (BiP) mRNA**

**One Way Repeated Measures Analysis of Variance**

**Data source:** Data 1 in Notebook 1 Grp78 (BiP) mRNA.JNB

**Normality Test (Shapiro-Wilk):**  Passed (P = 0.167)

**Equal Variance Test (Brown-Forsythe):** Passed (P = 0.142)

**Treatment Name N Missing Mean Std Dev SEM**

0 4 0 1.157 0.0329 0.0165

0.5 4 0 1.330 0.0241 0.0121

1 3 0 1.256 0.0560 0.0323

2 4 1 1.238 0.0378 0.0218

4 4 1 1.158 0.0560 0.0323

8 4 0 0.877 0.0139 0.00697

12 4 0 0.891 0.0618 0.0309

24 4 0 0.878 0.0857 0.0429

**Source of Variation DF SS MS F P**

Between Subjects 3 0.00777 0.00259

Between Treatments 7 0.919 0.131 50.583 <0.001

Residual 18 0.0467 0.00259

Total 28 0.984 0.0351

The differences in the mean values among the treatment groups are greater than would be expected by chance; there is a statistically significant difference (P = <0.001). To isolate the group or groups that differ from the others use a multiple comparison procedure.

Power of performed test with alpha = 0.050: 1.000

Expected Mean Squares:

Approximate DF Residual = 18.000

Expected MS(Subj) = var(res) + 7.000 var(Subj)

Expected MS(Treatment) = var(res) + var(Treatment)

Expected MS(Residual) = var(res)

Multiple Comparisons versus Control Group (Holm-Sidak method):

Overall significance level = 0.05

Comparisons for factor:

**Comparison Diff of Means t P P<0.050**

0 vs. 8 0.280 7.786 <0.001 Yes

0 vs. 24 0.279 7.736 <0.001 Yes

0 vs. 12 0.266 7.380 <0.001 Yes

0 vs. 0.5 0.173 4.794 <0.001 Yes

0 vs. 1 0.0919 2.339 0.090 No

0 vs. 2 0.0775 1.971 0.124 No

0 vs. 4 0.00604 0.154 0.880 No

**Figure 10B Grp94 mRNA**

**One Way Repeated Measures Analysis of Variance**

**Data source:** Data 1 in Notebook 1 Grp94 mRNA.JNB

**Normality Test (Shapiro-Wilk):**  Passed (P = 0.858)

**Equal Variance Test (Brown-Forsythe):** Failed (P < 0.050)

**Treatment Name N Missing Mean Std Dev SEM**

0 9 0 1.194 0.108 0.0362

0.25 8 0 1.003 0.625 0.221

.5 7 0 1.136 0.268 0.101

1 9 0 1.142 0.150 0.0500

2 8 0 1.082 0.189 0.0668

4 8 1 1.367 0.105 0.0398

8 6 2 0.926 0.364 0.182

**Source of Variation DF SS MS F P**

Between Subjects 8 1.397 0.175

Between Treatments 6 1.089 0.181 2.434 0.044

Residual 37 2.759 0.0746

Total 51 4.900 0.0961

The differences in the mean values among the treatment groups are greater than would be expected by chance; there is a statistically significant difference (P = 0.044). To isolate the group or groups that differ from the others use a multiple comparison procedure.

Power of performed test with alpha = 0.050: 0.488

Expected Mean Squares:

Approximate DF Residual = 37.000

Expected MS(Subj) = var(res) + 5.625 var(Subj)

Expected MS(Treatment) = var(res) + var(Treatment)

Expected MS(Residual) = var(res)

Multiple Comparisons versus Control Group (Holm-Sidak method):

Overall significance level = 0.05

Comparisons for factor:

**Comparison Diff of Means t P P<0.050**

0 vs. 8 0.399 2.338 0.140 No

0 vs. 0.25 0.206 1.534 0.512 No

0 vs. 4 0.196 1.396 0.528 No

0 vs. 2 0.128 0.952 0.722 No

0 vs. .5 0.110 0.784 0.684 No

0 vs. 1 0.0520 0.404 0.689 No

**Figure 10C Calreticulin mRNA**

**One Way Repeated Measures Analysis of Variance**

**Data source:** Data 1 in Notebook1 calreticulin mRNA

**Normality Test (Shapiro-Wilk):**  Passed (P = 0.283)

**Equal Variance Test (Brown-Forsythe):** Passed (P = 0.263)

**Treatment Name N Missing Mean Std Dev SEM**

0 4 0 0.667 0.0769 0.0385

.25 4 0 0.745 0.0773 0.0387

.5 3 0 0.602 0.00687 0.00396

1 4 0 0.786 0.136 0.0679

2 4 0 0.823 0.0404 0.0202

4 5 0 0.992 0.240 0.108

8 4 0 1.014 0.0601 0.0300

24 4 0 0.850 0.0658 0.0329

**Source of Variation DF SS MS F P**

Between Subjects 4 0.112 0.0281

Between Treatments 7 0.608 0.0869 7.274 <0.001

Residual 20 0.239 0.0119

Total 31 0.917 0.0296

The differences in the mean values among the treatment groups are greater than would be expected by chance; there is a statistically significant difference (P = <0.001). To isolate the group or groups that differ from the others use a multiple comparison procedure.

Power of performed test with alpha = 0.050: 0.994

Expected Mean Squares:

Approximate DF Residual = 20.000

Expected MS(Subj) = var(res) + 6.000 var(Subj)

Expected MS(Treatment) = var(res) + var(Treatment)

Expected MS(Residual) = var(res)

Multiple Comparisons versus Control Group (Holm-Sidak method):

Overall significance level = 0.05

Comparisons for factor:

**Comparison Diff of Means t P P<0.050**

0 vs. 4 0.386 4.992 <0.001 Yes

0 vs. 8 0.348 4.109 0.003 Yes

0 vs. 24 0.184 2.170 0.194 No

0 vs. 2 0.156 1.847 0.283 No

0 vs. 1 0.119 1.405 0.439 No

0 vs. .25 0.0783 0.925 0.598 No

0 vs. .5 0.0477 0.524 0.606 No

**Figure 10D XP1 mRNA**

**One Way Repeated Measures Analysis of Variance**

**Data source:** Data 1 in Notebook 1 XBP1 mRNA.JNB

**Normality Test (Shapiro-Wilk):**  Passed (P = 0.950)

**Equal Variance Test (Brown-Forsythe):** Passed (P = 0.616)

**Treatment Name N Missing Mean Std Dev SEM**

0 5 0 1.627 0.464 0.207

.5 3 0 1.668 0.115 0.0663

1 4 0 1.348 0.421 0.210

2 3 0 1.821 0.606 0.350

4 4 0 1.187 0.280 0.140

8 5 0 1.243 0.441 0.197

12 3 0 1.266 0.188 0.108

24 4 0 4.022 0.928 0.464

**Source of Variation DF SS MS F P**

Between Subjects 4 1.887 0.472

Between Treatments 7 24.363 3.480 16.818 <0.001

Residual 19 3.932 0.207

Total 30 30.462 1.015

The differences in the mean values among the treatment groups are greater than would be expected by chance; there is a statistically significant difference (P = <0.001). To isolate the group or groups that differ from the others use a multiple comparison procedure.

Power of performed test with alpha = 0.050: 1.000

Expected Mean Squares:

Approximate DF Residual = 19.000

Expected MS(Subj) = var(res) + 5.750 var(Subj)

Expected MS(Treatment) = var(res) + var(Treatment)

Expected MS(Residual) = var(res)

Multiple Comparisons versus Control Group (Holm-Sidak method):

Overall significance level = 0.05

Comparisons for factor:

**Comparison Diff of Means t P P<0.050**

0 vs. 24 2.404 7.668 <0.001 Yes

0 vs. 4 0.431 1.374 0.708 No

0 vs. 8 0.384 1.334 0.668 No

0 vs. 12 0.336 0.975 0.812 No

0 vs. 1 0.269 0.859 0.785 No

0 vs. 2 0.219 0.633 0.783 No

0 vs. .5 0.0650 0.188 0.853 No

**Figure 12 Hrd1 mRNA**

**One Way Repeated Measures Analysis of Variance**

**Data source:** Data 1 in Notebook1 Hrd1 mRNA

**Normality Test (Shapiro-Wilk):**  Passed (P = 0.898)

**Equal Variance Test (Brown-Forsythe):** Passed (P = 0.055)

**Treatment Name N Missing Mean Std Dev SEM**

0 6 0 1.045 0.220 0.0898

0.25 5 0 1.317 0.249 0.111

.5 5 0 0.907 0.193 0.0864

1 4 0 0.965 0.145 0.0725

2 4 0 0.717 0.0979 0.0489

4 4 0 0.916 0.162 0.0810

8 4 0 0.786 0.107 0.0535

24 3 0 0.687 0.239 0.138

**Source of Variation DF SS MS F P**

Between Subjects 5 0.132 0.0264

Between Treatments 7 1.329 0.190 5.059 0.002

Residual 22 0.826 0.0375

Total 34 2.231 0.0656

The differences in the mean values among the treatment groups are greater than would be expected by chance; there is a statistically significant difference (P = 0.002). To isolate the group or groups that differ from the others use a multiple comparison procedure.

Power of performed test with alpha = 0.050: 0.940

Expected Mean Squares:

Approximate DF Residual = 22.000

Expected MS(Subj) = var(res) + 5.400 var(Subj)

Expected MS(Treatment) = var(res) + var(Treatment)

Expected MS(Residual) = var(res)

Multiple Comparisons versus Control Group (Holm-Sidak method):

Overall significance level = 0.05

Comparisons for factor:

**Comparison Diff of Means t P P<0.050**

0 vs. 24 0.438 3.016 0.044 Yes

0 vs. 2 0.395 2.988 0.040 Yes

0 vs. 8 0.327 2.473 0.103 No

0 vs. 0.25 0.222 1.809 0.297 No

0 vs. .5 0.189 1.540 0.359 No

0 vs. 4 0.197 1.488 0.279 No

0 vs. 1 0.148 1.118 0.276 No

**Figure 12B EDEM1 mRNA**

**One Way Repeated Measures Analysis of Variance**

**Data source:** Data 1 in Notebook 1 EDEM1 mRNA.JNB

**Normality Test (Shapiro-Wilk):**  Passed (P = 0.409)

**Equal Variance Test (Brown-Forsythe):** Passed (P = 0.790)

**Treatment Name N Missing Mean Std Dev SEM**

0 6 0 1.240 0.255 0.104

0.25 4 0 1.540 0.147 0.0734

0.5 4 0 1.495 0.226 0.113

1 5 0 1.510 0.216 0.0966

2 4 0 1.887 0.380 0.190

4 4 0 1.912 0.233 0.117

8 4 0 1.355 0.217 0.109

24 4 0 2.607 0.596 0.298

**Source of Variation DF SS MS F P**

Between Subjects 5 0.187 0.0373

Between Treatments 7 5.146 0.735 6.890 <0.001

Residual 22 2.348 0.107

Total 34 8.288 0.244

The differences in the mean values among the treatment groups are greater than would be expected by chance; there is a statistically significant difference (P = <0.001). To isolate the group or groups that differ from the others use a multiple comparison procedure.

Power of performed test with alpha = 0.050: 0.992

Expected Mean Squares:

Approximate DF Residual = 22.000

Expected MS(Subj) = var(res) + 5.400 var(Subj)

Expected MS(Treatment) = var(res) + var(Treatment)

Expected MS(Residual) = var(res)

Multiple Comparisons versus Control Group (Holm-Sidak method):

Overall significance level = 0.05

Comparisons for factor:

**Comparison Diff of Means t P P<0.050**

0 vs. 24 1.306 5.799 <0.001 Yes

0 vs. 4 0.611 2.713 0.074 No

0 vs. 2 0.585 2.599 0.079 No

0 vs. 0.25 0.239 1.062 0.760 No

0 vs. 1 0.217 1.051 0.664 No

0 vs. 0.5 0.194 0.859 0.639 No

0 vs. 8 0.0538 0.239 0.813 No
