# Supplementary material for: Activation of the EIF2α/ATF4 and ATF6 Pathways in DU-145 Cells by Boric Acid at the Concentration Reported in Men at the US Mean Boron Intake
Source: Biol Trace Elem Res. 2016 Sep 1;176(2):278–93. doi: 10.1007/s12011-016-0824-y (PMC5344959; doi:10.1007/s12011-016-0824-y)
Supplement: Supplementary file 2 — (DOCX 19 kb) [file 12011_2016_824_MOESM2_ESM.docx]

**Supplement 2**

**ATCC Description of DU-145 Karyotype**

“This is a hypotriploid human cell line. Both 61 and 62 chromosome numbers had the highest rate of occurrence in 30 metaphase counts. The rate of higher ploides was 3%. The t(11q12q), del(11)q23), 16q+, del(9)(p11),del(1)(p32) and 6 other marker chromosomes were found in most cells. The N13 was usually absent. The Y chromosome is abnormal through translocation to an unidentified chromosomal segment. The X chromosome was present in single copy.” (87)

**COSMIC Ploidy for DU-145**

The average ploidy for DU-145 is 2.66. COSMIC uses average ploidy > 2.7 to define genome duplication. A gain in average genome ploidy <=2.7 AND total copy number > = 5 OR average genome ploidy > 2.7 AND total copy number >=9. (36)

| \| **Table S2-1** \| **COSMIC Cell Line DU-145: Search for Variants of Measured Genes** \| \| \| \|  \|  \| \| \| --- \| --- \| --- \| --- \| --- \| --- \| --- \| --- \| \|  \|  \|  \|  \|  \| \| \|  \| \|  \| \| **Gene^1^** \| **Approved Symbol** \| **Approved Name** \| **HGNC ID** \| **Mutations** \| \| \|  \| \|  \| \|  \|  \|  \|  \|  \| \| \|  \| \|  \| \| ATF4 \| ATF4 \| activating transcription factor 4 \| HGNC:786 \| substitution \| \| \|  \| \|  \| \| ATF6A \| ATF6A \| activating transcription factor 6A \| HGNC:791 \| no record \| \| \|  \| \|  \| \| Calreticulin \| CALR \| calreticulin \| HGNC:1455 \| no record \| \| \|  \| \|  \| \| EDEM1 \| EDEM1 \| ER degradation enhancer, mannosidase alpha-like 1 \| HGNC:18967 \| no record \| \| \|  \| \|  \| \| Eif2 alpha \| EIF2A \| eukaryotic translation initiation factor 2A \| HGNC:3254 \| no record \| \| \|  \| \|  \| \| GADD153 \| DDIT3 \| DNA damage inducible transcript 3 \| HGNC:2726 \| no record \| \| \|  \| \|  \| \| GADD34 \| [PPP1R15A](https://grch37-cancer.sanger.ac.uk/cosmic/gene/analysis?ln=PPP1R15A) \| protein phosphatase 1 regulatory subunit 15A \| HGNC:14375 \| no record \| \| \|  \| \|  \| \| GAPDH \| GAPDH \| glyceraldehyde-3-phosphate dehydrogenase \| HGNC:4141 \| no record \| \| \|  \| \|  \| \| GRP78 (BiP) \| HSPA5 \| heat shock protein family A (Hsp70) member 5 \| HGNC:5238 \| no record \| \| \|  \| \|  \| \| GRP94 \| HSP90B1 \| heat shock protein 90kDa beta family member 1 \| HGNC:12028 \| no record \| \| \|  \| \|  \| \| HERP \| HERPUD1 \| homocysteine-inducible, endoplasmic reticulum \| HGNC:13744 \| no record \| \| \|  \| \|  \| \|  \|  \| stress-inducible, ubiquitin-like domain member 1 \|  \|  \| \| \|  \| \|  \| \| HRD1 \| SYVN1 \| synoviolin 1 \| HGNC:20738 \| no record \| \| \|  \| \|  \| \| IRE1 \| ERN1 \| endoplasmic reticulum to nucleus signaling 1 \| HGNC 3449 \| substitution \| \| \|  \| \|  \| \| XBP1 \| XBP1 \| X-box binding protein 1 \| HGNC:12801 \| no record \| \| \|  \| \|  \| \|  \|  \|  \|  \|  \| \| \|  \| \|  \| \| ^1^ HGNC gene nomenclature committee  http://www.genenames.org/cgi-bin/search?search_type=all&search=ire1&submit=Submit \| \| \| \| \| \| \| \| \| \| |  | | |  |  |
| --- | --- | --- | --- | --- | --- | --- | --- | --- | --- | --- | --- | --- | --- | --- | --- | --- | --- | --- | --- | --- | --- | --- | --- | --- | --- | --- | --- | --- | --- | --- | --- | --- | --- | --- | --- | --- | --- | --- | --- | --- | --- | --- | --- | --- | --- | --- | --- | --- | --- | --- | --- | --- | --- | --- | --- | --- | --- | --- | --- | --- | --- | --- | --- | --- | --- | --- | --- | --- | --- | --- | --- | --- | --- | --- | --- | --- | --- | --- | --- | --- | --- | --- | --- | --- | --- | --- | --- | --- | --- | --- | --- | --- | --- | --- | --- | --- | --- | --- | --- | --- | --- | --- | --- | --- | --- | --- | --- | --- | --- | --- | --- | --- | --- | --- | --- | --- | --- | --- | --- | --- | --- | --- | --- | --- | --- | --- | --- | --- | --- | --- | --- | --- | --- | --- | --- | --- | --- | --- | --- | --- | --- | --- | --- | --- | --- | --- | --- | --- | --- | --- | --- | --- | --- | --- | --- | --- | --- | --- | --- | --- | --- | --- | --- | --- | --- | --- | --- | --- | --- | --- | --- | --- | --- | --- | --- | --- | --- | --- | --- | --- | --- | --- | --- | --- | --- | --- | --- | --- | --- | --- | --- | --- | --- | --- | --- | --- | --- | --- | --- | --- | --- | --- | --- | --- | --- | --- | --- | --- | --- | --- | --- | --- | --- |
|  |  |  | |  |  |
|  |  |  |  |  | |
|  |  |  |  |  | |
|  |  |  |  |  | |
|  |  |  |  |  | |

| **Table S2-2 ATF4 Mutation Record in DU-145 from COSMIC Catalogue of Somatic Mutations** | |
| --- | --- |
|  |  |
| **ATF Mutation** | **COSMIC Record** |
|  |  |
| ATF4 Chromosome Position (ensembl) | 22:39521446-39522602 |
| Census Gene | No |
| Transcript with Mutation | ENST0000396680 |
| Amino Acid Mutation | Unknown |
| Coding DNA Sequence (CDS) Mutation | c.226+9G>A |
| Somatic Status | Unknown |
| Zygosity | Heterozygous |
| Validated | Unverified |
| Type | Substitution - intronic |
| Chromosome Position of Mutation | 22:38521680 |
| Copy Number Analysis (CONAN) | No data available |
| ATF4 Regulatory Feature -Promoter | Start 22:39519154; End 22:39522954 |

| **Table S2-3** | **COSMIC Record of ATF4 Mutations in Prostate Tissue** | | |
| --- | --- | --- | --- |
|  |  |  |  |
| **Histology** | **Proportion ATF4 Mutation** | **Copy Number** | **Gene Expression** |
| Normal | not reported | not reported | not reported |
| Carcinoma | 16.67% | not reported | under-expressed 16.67% |
| Hyperplasia | not reported | not reported | not reported |

| **Table S2-4 IRE1 (ERN1) Mutation Record in DU-145 from COSMIC Catalogue of Somatic Mutations** | |  |
| --- | --- | --- |
|  |  |  |
| **IRE1 Mutation** | **COSMIC Record** |  |
|  |  |  |
| IRE1 (ERN1) Chromosome Position (ensembl) | 17:64,043,988-64,130,029 |  |
| Census Gene | No |  |
| Transcript with Mutation | ENST00000433197 |  |
| Amino Acid Mutation | Unknown |  |
| Coding DNA Sequence (CDS) Mutation | c.2183G>T |  |
| Somatic Status | Unknown |  |
| Zygosity | Heterozygous |  |
| Validated | Unverified |  |
| Type | Substitution - missense |  |
| Chromosome Position of Mutation | 17:64052850 |  |
| Copy Number Analysis (CONAN) | no data available |  |

| **Table S2-5 IRE1 (ERN1) Ensemble Regulatory Build** | |  |  |  |
| --- | --- | --- | --- | --- |
|  |  |  |  |  |
| **Reg. Region** | **Type** | **Location** |  |  |
| ENSR00001894356 | Enhancer | 17:64050764-64051800 | | |
| ENSR00001894357 | Enhancer | 17:64055964-64056564 | | |
| ENSR00001894358 | Enhancer | 17:64050764-64051800 | | |
| ENSR00001894357 | Enhancer | 17:64055964-64056564 | | |
| ENSR00001894358 | Enhancer | 17:64056964-64057363 | | |
| ENSR00001894359 | Enhancer | 17:64058164-64058563 | | |
| ENSR00001894360 | Promoter Flanking Region | 17:64059602-64064562 | | |
| ENSR00001351069 | CTCF binding site | 17:64061364-64061763 | | |
| ENSR00001351071 | CTCF binding site | 17:64063564-64064163 | | |
| ENSR00001894361 | Enhancer | 17:64065801-64066400 | | |
| ENSR00001894362 | Enhancer | 17:64066801-64068000 | | |
| ENSR00001894363 | Promoter Flanking Region | 17:64072802-64078600 | | |
| ENSR00001894364 | Enhancer | 17:64079164-64079963 | | |
| ENSR00001894364 | Promoter Flanking Region | 17:64080202-64086999 | | |
| ENSR00001640387 | CTCF binding site | 17:64081764-64082163 | | |
| ENSR00001894366 | Enhancer | 17:64087201-64089200 | | |
| ENSR00001894367 | Enhancer | 17:64089601-64094000 | | |
| ENSR00001894368 | Promoter Flanking Region | 17:64094802-64098562 | | |
| ENSR00001894369 | Promoter Flanking Region | 17:64102802-64188199 | | |
| ENSR00001640389 | CTCF binding site | 17:64106564-64106963 | | |
| ENSR00001640390 | CTCF binding site | 17:64107764-64108163 | | |
| ENSR00001351087 | CTCF binding site | 17:64113164-64113563 | | |
| ENSR00001894370 | Enhancer | 17:64122364-64122763 | | |
| ENSR00001894371 | Enhancer | 17:64123964-64124363 | | |
| ENSR00001640395 | CTCF binding site | 17:64125964-64126363 | | |
| ENSR00001894372 | Promoter | 17:64128200-64130801 | | |

| **Table S2-6** | **COSMIC Record of IRE1 (ERN1) Mutations in Prostate Tissue** | | |
| --- | --- | --- | --- |
|  |  |  |  |
| **Tissue Histology** | **Proportion IRE1 (ERN1) Mutation** | **Copy Number** | **Gene Expression** |
| Normal | 0 of 1 tested | not reported | not reported |
| Carcinoma | 0 of 1070 tested | not reported | not reported |
| Hyperplasia | 0 of 1 tested | not reported | not reported |
| Adenoma | 0 of 63 tested | not reported | not reported |
| PIN | 0 of 10 tested | not reported | not reported |
